# Changes in DNA damage, molecular integrity, and copy number for plastid DNA and mitochondrial DNA during maize development

**DOI:** 10.1093/jxb/eru359

**Published:** 2014-09-26

**Authors:** Rachana A. Kumar, Delene J. Oldenburg, Arnold J. Bendich

**Affiliations:** Department of Biology, University of Washington, Seattle, WA 98195-5325, USA

**Keywords:** DNA damage, DNA repair, organellar DNA, PCR, reactive oxygen species, *Zea mays*.

## Abstract

The amount and structural integrity of organellar DNAs change during plant development, although the mechanisms of change are poorly understood. Using PCR-based methods, we quantified DNA damage, molecular integrity, and genome copy number for plastid and mitochondrial DNAs of maize seedlings. A DNA repair assay was also used to assess DNA impediments. During development, DNA damage increased and molecules with impediments that prevented amplification by *Taq* DNA polymerase increased, with light causing the greatest change. DNA copy number values depended on the assay method, with standard real-time quantitative PCR (qPCR) values exceeding those determined by long-PCR by 100- to 1000-fold. As the organelles develop, their DNAs may be damaged in oxidative environments created by photo-oxidative reactions and photosynthetic/respiratory electron transfer. Some molecules may be repaired, while molecules with unrepaired damage may be degraded to non-functional fragments measured by standard qPCR but not by long-PCR.

## Summary

During development in maize, chloroplast and mitochondrial DNA damage increases, copy number of unimpeded DNA molecules decreases, and *in vitro* DNA repair increases, with light causing the greatest change.

## Introduction

The number of copies of the genome in the nucleus is two for most somatic cells in diploid plants and animals, regardless of developmental or environmental changes. For mitochondria and plastids, however, there are multiple copies of the genome, and copy number changes greatly during development. For example, human mitochondrial DNA (mtDNA) copy number varies from about 1000 to 10 000 among tissues, with large differences among individual persons (Frahm *et al.*, 2005). Furthermore, whereas nuclear DNA is stable during development, mtDNA is degraded and turns over in animals and plants (Bendich, 2010). Plant cells contain both mtDNA and plastid DNA (ptDNA), and copy numbers can change independently (Kuroiwa, 1991; Woloszynska, 2010). During development in maize, for example, organellar DNA (orgDNA) copy numbers can change 20-fold, with light differentially affecting both increases and decreases among different tissues (Oldenburg *et al.*, 2006, 2013; Zheng *et al.*, 2011). Even in the single-celled alga *Euglena* under constant growth conditions, both ptDNA and mtDNA are extremely unstable (half-lives of 1.6 and 1.8 cell doublings, respectively), whereas nuclear DNA turnover could not be detected (Manning and Richards, 1972; Richards and Ryan, 1974).

Why does orgDNA copy number vary so greatly? In an early proposal, high copy number reflects an increased demand for organellar ribosomes that can only be satisfied by the increased rRNA gene number that results from genome amplification (Bendich, 1987). More recently, an additional reason for copy number change emerged that also explained the turnover of mtDNA. If human mtDNA was damaged but not repaired, those damaged molecules were degraded to avoid mutation (Shokolenko *et al.*, 2009; Liu and Demple, 2010).

The frequency and repair of damage in mtDNA have been studied in animals and yeast by treatment with genotoxic agents (such as H_2_O_2_) or using mutants in repair functions and then measuring the resulting additional DNA lesions per 10kb of DNA (Hunter *et al.*, 2010). These assays showed increases in DNA lesions following treatment but did not evaluate differences in mtDNA damage among tissues. Similarly, more damage to ptDNA in *Arabidopsis* was reported for a mutant in the DNA polymerase protein PolI compared with the wild type (Parent *et al.*, 2011). DNA sustains damage mainly by oxidative and hydrolytic processes *in vivo* due to the presence of reactive oxygen species (ROS) and water. Lesions due to oxidative processes include 8-oxo-G, ring-saturated pyrimidines (e.g. thymine glycol, cytosine hydrates), and lipid peroxidation adducts [pyrimido(1,2-*a*)purin-10(*3H*)-one, etheno-DNA adducts]. Hydrolytic lesions include depurination, depyrimidination, and cytosine deamination (Table 1). In addition to these endogenous lesions, others may be caused by environmental factors like UV light (pyrimidine dimers). Although the DNA repair mechanisms operating in plant organelles are still poorly understood, it is likely that some lesions may be rectified. For example, in *Arabidopsis*, the base excision repair pathway may remove oxidative lesions, such as thymine glycol (Gutman and Niyogi, 2009). In addition, proteomic analysis of maize plastids revealed developmental changes in DNA-associated proteins, including repair enzymes, likely to affect orgDNA copy number (Majeran *et al.*, 2012).

**Table 1.** DNA impediments and their impact on long-PCR and repair assays

Impediment^*a*^	Examples	Causes	Repair by PreCR^*b*^
*Lesions*			
Pyrimidine dimer	Thymine dimer	UV light	Yes^*c*^
	Cytosine dimer	UV light	Yes^*c*^
Abasic site	Depurination	Hydrolysis	Yes
	Depyrimidination		Yes
Oxidized bases	8-oxo-G^*d*^	Oxidation	Yes
	8-oxo-A		No
	Thymine glycol		Yes
Deaminated cytosine		Hydrolysis	Yes
Bulky adduct	Benzo[a]pyrene diol epoxide-dG		ND
	M1dG	Lipid peroxidation	ND
*Discontinuity*			
Single-strand break	Nick	Hydrolysis	Yes
		Endonuclease	
		Topoisomerase	
	Single-strand gap	Replication	Yes; 5–10 nt
		Transcription	
Double-strand break	Fragmentation	Hydrolysis	No^*c*^
		Endonuclease	
		Topoisomerase	
Branch point	Replication fork		ND
	Recombination junction		
Protein-DNA crosslinks		Topoisomerase	No
		Formaldehyde	

8-oxo-G, 8-oxo-7,8-dihydro-2’-deoxyguanine; 8-oxo-A, 8-oxo-7,8-dihydro-2’-deoxyadenine; M1dG, pyrimido[1,2-*a*]purin-10(*3H*)-one; ND, not determined.

^*a*^ Types, examples, and causes of impediments are from Friedberg *et al.* (2006).

^*b*^ NEB product website (https://www.neb.com/tools-and-resources/usage-guidelines/dna-damage-and-precr) and/or Tom Evans (product scientist for NEB)

^*c*^ See Supplementary Material 3.

^*d*^ Except for 8-oxo-G, all other impediments may inhibit LongAmp polymerase.

Various procedures have been used to assess changes in DNA copy number during development: (i) measuring the increase in the rate of probe DNA strand reassociation caused by the addition of a large amount of DNA extracted from total tissue (Lamppa and Bendich, 1979; Lamppa and Bendich, 1984); (ii) blot hybridization of a probe to restriction-digested total tissue DNA (ttDNA) (Li *et al.*, 2006; Zheng *et al.*, 2011; Udy *et al.*, 2012; Oldenburg *et al.*, 2013); (iii) fractionation of orgDNA by pulsed-field gel electrophoresis (PFGE) (Oldenburg *et al.*, 2006, 2013; Shaver *et al.*, 2006); (iv) quantitative fluorescence using a DNA-specific fluorophore and either intact cells or organelles isolated from cells (Oldenburg and Bendich, 2004*a*; Rowan *et al.*, 2004; Shaver *et al.*, 2006; Oldenburg *et al.*, 2013); and (v) real-time quantitative PCR (qPCR) (Zoschke *et al.*, 2007; Rowan *et al.*, 2009; Preuten *et al.*, 2010; Udy *et al.*, 2012). These procedures should yield equivalent results providing that the size and molecular integrity of the DNA molecules are maintained, as is the case for chromosomal DNA in the nucleus. For orgDNAs, however, molecular integrity declines sharply during leaf development, so these procedures can yield conflicting results (Day and Madesis, 2007; Rowan and Bendich, 2009; Oldenburg *et al.*, 2014).

Here, we used qPCR and long-PCR assays to assess orgDNA damage and genome copy number as maize seedlings developed under light, dark-to-light, and dark growth conditions. We also developed a new method, ‘molecular integrity PCR’ (miPCR), to quantify long orgDNA molecules without DNA impediments that would (presumably) interfere with the coding and inheritance functions of the DNA. In addition, an *in vitro* DNA repair assay was used that confirmed the presence of DNA impediments, including those associated with oxidative processes. As the seedlings developed, damage to orgDNA increased in dark-grown and dark-to-light-transferred plants, whereas high damage levels were found in light-grown plants. In addition, the level of ‘functional’ orgDNA (as measured by miPCR) decreased in dark- and dark-to-light-grown plants and remained at a low level for light-grown plants. Remarkably, the levels of such functional orgDNA were greatly reduced compared with total copies as measured by standard qPCR. This finding could be attributed to the inability of qPCR to distinguish between intact and fragmented forms of orgDNA, thus inflating estimates of genome copy number. Overall, light affected both damage and levels of functional DNA in both plastids and mitochondria, even though mitochondria have no known photoreceptors. We surmise that functional ptDNA is maintained as required for chloroplast development, but soon after greening the DNA may be damaged by ROS and subsequently degraded. Functional mtDNA is also preserved prior to photosynthesis but may no longer be needed in mature green cells of maize.

## Materials and methods

### Plant tissue and DNA isolation

*Zea mays* (inbred line B73) seeds were imbibed overnight and sown in Sunshine soil Mix #4. The seedlings were grown for 13 d with a 16h light/8h dark photoperiod, in continuous dark or for 12 d in dark followed by 1 d in light. The light intensity was ~500 µmol s^–1^ m^–2^. Seedlings were washed with 0.5% sarkosyl for ~3min and then rinsed with distilled water. Tissue was harvested from 20–25 plants as follows: base of stalk (S1: 5mm above the node); top of stalk (S2: 5mm below the ligule of the first leaf), entire first and second leaf blades (L1 and L2, respectively). Stalk tissue was composed of several concentric rings of leaves, the outermost being the first leaf sheath. L1 was the fully expanded blade, whereas L2 was still developing, and for L2 tissue the unexpanded leaf blade above the L1 ligule was harvested. Five independent sets of plants were grown under the three growth conditions and the tissues were harvested, representing five biological replicates. Each biological replicate comprised 12 samples (four tissues×three growth conditions) and a total of 60 samples was analysed (12 tissues×five biological replicates). TtDNA was extracted using cetyltrimethylammonium bromide (CTAB) as described by Rogers and Bendich (1988). Tissue was frozen in liquid nitrogen and ground to a powder with dry ice. An equal volume of 2× CTAB buffer [2% CTAB (w/v), 100mM Tris/HCl (pH 8.0), 20mM EDTA, 1.4M NaCl, 1% polyvinylpyrrolidone (*M*_r_ 40 000; w/v); preheated to 65 °C] was added to the frozen powder and incubated at 65 °C for 30min. Phenol was not used in the procedure. After chloroform extraction and isopropanol precipitation, the DNA was suspended in 10mM Tris/HCl (pH 8), 1mM EDTA (TE buffer), and DNA integrity was assessed by agarose gel electrophoresis. The ttDNA was quantified using a Quant-it kit (Life Technologies, NY, USA), diluted to 3ng μl^–1^, and 6 and 15ng of DNA was used for the qPCR and long-PCR assays, respectively.

For in-gel preparation of ptDNA, plastids from entire stalk and L1 from light-grown 14-d-old plants were isolated using high-salt buffer and purified using Percoll (Oldenburg *et al.*, 2006). Isolated plastids were embedded in low-melting-point agarose (Oldenburg and Bendich, 2004*a*). In-gel plastids were soaked in lysis buffer [40mM EDTA (pH 8), 1% sarkosyl, 200 µg ml^–1^ of proteinase K] overnight at 48 °C. The proteinase K was inactivated with phenylmethylsulfonyl fluoride, and the plugs were washed four times with TE buffer. ptDNA in the lysis buffer and first TE wash (eluates) was extracted using Geneclean II (Qbiogene). Using qPCR and the standards described below, absolute quantification of ptDNA copies was determined for the lysis buffer, eluates, and fraction retained in-gel (after melting the gel plugs at 75 °C).

### DNA damage assay, miPCR, and determination of orgDNA without structural impediments

Fragments of 11 207 and 11 164bp were amplified using LongAmp *Taq* (New England Biolabs, MA, USA) from ptDNA and mtDNA, respectively. The assay was optimized for cycle number to be in the exponential phase, with primer efficiencies of ~100%, and for orgDNA-specific primers for accurate quantification of long orgDNA copies (Supplementary Figs S1–S7 in Supplementary Material 1 at *JXB* online).

The primers used for long-PCR amplification were: *nad4*, 11 164bp mtDNA-specific (nad4_F3, 5′-GTTGGACCACAGGCAAAAGT-3′, and trnk_R1, 5′-GCGAGGAATGGAAGCAGTAG-3′); *rps14*, 11 207bp ptDNA-specific (rps14_F1, 5′-ATCTTGTTGCACCCGGTAAC-3′, and rps14_R5, 5′-TATCCTGACCCTTTCTTGTGC-3′). The reaction mix contained 1× NEB LongAmp buffer with 1.9mM (for ptDNA) or 1.6mM (for mtDNA) MgSO_4_, 200nM dNTP, 400nM of each primer, 2.5U of NEB LongAmp enzyme and 15ng ttDNA in a total volume of 50 µl. For long-PCR, the following cycling program was used: one cycle of 94 °C for 30 s, 20 (for ptDNA) or 22 (for mtDNA) cycles of 94 °C for 30 s, 65 °C (for ptDNA) or 61 °C (for mtDNA) for 1min, and 65 °C for 7.2min, followed by a final extension at 65 °C for 10min. The long-PCR products were fractionated by agarose gel electrophoresis along with 0.75–37.5ng of DNA standards (MassRuler DNA; Fermentas, USA) that formed a ‘standard curve’ for the absolute quantification of orgDNA (Supplementary Fig. S7). The band intensity was determined using NIH ImageJ (Schneider *et al.*, 2012).The nanograms of DNA in each long-PCR band was converted to end-point PCR copy number. The number of initial template DNA copies was then determined from end-point PCR (for example, at the end of 20 cycles) and the amplification factor as: end-point copies/2^number of PCR cycles^.

Template copies thus calculated were normalized to the orgDNA copy number as determined by qPCR (see below): long-PCR orgDNA copies/qPCR orgDNA copies. The sample with the highest value was considered the least damaged, set at 1, and used to determine the relative amplification for the other samples. For example, if the highest normalized value among samples was 0.5, this was set at 1, and the relative amplification would be 0.5 for another sample that had a normalized value of 0.25. Finally, orgDNA impediments per 10kb was determined using the formula: [–ln (relative amplification)×10 000 bp]/amplicon size (bp). Four to six technical replicates per sample were used for DNA damage assays. Template copies for orgDNA without impediments (unimpeded orgDNA) were then normalized to qPCR-determined orgDNA copies and multiplied by 100 to give a percentage (Supplementary Table S1 in Supplementary Material 2 at *JXB* online). Template orgDNA copies were also normalized to a single-copy nuclear DNA gene (*adh1* copies determined by qPCR, described below) to obtain miPCR copies (Supplementary Table S1).

### Real-time qPCR

Primers to amplify orgDNA and not NUPTs/NUMTs were designed (Kumar and Bendich, 2011). The primers used for qPCR amplification were as follows: *adh1*, 156bp nucDNA-specific (adh1_left, 5′-GCTCCTCACAGGCTCATCTC-3′, and adh1_right, 5′-AGGCGGACCTTTGCACTT-3′); *rps14*, 127bp ptDNA-specific (rps14_F1, 5’-ATCTTGTTGCACCCGGTAAC-3′, and rps14_R2, 5′-CCTACACGCCTTCATCGACGTT-3′); and *nad4*, 187bp mtDNA-specific (nad4_F2, 5′-GCAAAAGTCCTTCCAC GGCA-3′, and nad4_R1, 5′-AGCAAGCGTAGGCAA CCAAAC-3′). The qPCRs contained 1× iQ™ SYBR Green Supermix®, 400nM of each primer, and 6ng ttDNA in a total volume of 25 µl. A Chromo4™ thermal cycler (Bio-Rad, CA, USA) was used for qPCR using the following cycling program: one cycle of 94 °C for 3.3min, and 40 cycles of 94 °C for 15 s, 59 °C for 15 s, and 72 °C for 20 s. qPCR data from six technical replicates were analysed using Opticon Monitor™ software. Melting curves from 65 to 95 °C were analysed to confirm the presence of a single product. qPCR efficiencies for the primer sets were between 1.9 and 2.1. The ptDNA and mtDNA copies per haploid nuclear genome were determined using absolute quantification with DNA standards produced using maize ttDNA template and the three primer sets. The concentration of the DNA standards was determined using the Quant-it assay. The range of copies was 100 to 10 000 µl^–1^ for *adh1*, 10 000 to 1 000 000 copies µl^–1^ for *rps14*, and 1000 to 100 000 copies µl^–1^ for *nad4*. We followed the MIQE guidelines for qPCR (Bustin *et al.*, 2009).

### DNA repair

In order to assess DNA damage and repairable lesions, orgDNAs were treated with repair enzymes, followed by long-PCR. PreCR Repair mix (New England Biolabs, MA, USA) contains seven enzymes that can repair most DNA lesions, including abasic sites, nicks, thymidine dimers, blocked 3’-ends, oxidized guanine, oxidized pyrimidines, and deaminated cytosine. Specifically, the mix contains enzymes for repair of oxidative damage [endonuclease IV, endonuclease VIII, formamidopyrimidine-DNA glycosylase], UV damage (T4 pyrimidine dimer glycosylase), and hydrolytic damage (uracil-DNA glycosylase). It also contains DNA ligase and polymerase to complete the repair process. However, the repair mix will not mend protein–DNA crosslinks or join double-strand breaks (Supplementary Fig. S10 in Supplementary Material 3 at *JXB* online). First, 200ng of ttDNA was combined with 1× ThermoPol Buffer, 100 µM dNTPs, and 1× NAD^+^ in a 98 µl reaction volume. This mixture was divided into two equal volumes. Then, 1 µl of PreCR Repair mix was added to one tube (+Repair) and 1 µl of water to the other tube (–Repair). The +Repair tube was incubated at 37 °C for 15min. The +/– Repair mixtures then served as template for the long-PCR, as described above, and the products were fractionated by agarose gel electrophoresis. ImageJ software was used to compare the pixel levels of the ethidium bromide-stained DNA fluorescence between +Repair and –Repair PCR products and the fold change was calculated.

### Statistical analysis

Statistical analyses were performed on the data obtained by qPCR, long-PCR (% unimpeded long orgDNA copies), and miPCR for five biological replicates (Supplementary Tables S2–S6 in Supplementary Material 4 at *JXB* online). The analysis of variance (ANOVA) model was applied to assess differences among tissues during development, entire seedlings under three growth conditions, and the combination of both (interaction). If ANOVA revealed a significant *p* value, then Tukey’s honestly significant difference (HSD) test for multiple comparisons was used to identify each pair of samples exhibiting a significant difference (Supplementary Tables S2–S6). Statistical analyses were not performed on the data obtained for impediments per 10kb because these are relative values that were determined independently for each biological replicate. Thus, the values for all the replicates could not be combined. Instead, normalized values (long-PCR orgDNA copies/qPCR orgDNA copies) for the replicates were averaged, and a single value of impediments per 10kb was determined for each tissue.

The relationship between ptDNA and mtDNA was evaluated for these parameters: (i) copy number from qPCR; (ii) copy number from miPCR; (iii) percentage of unimpeded orgDNA; and (iv) orgDNA impediments per 10kb (Supplementary Material 5 at *JXB* online). The linear or logarithmic regression model was employed to obtain *R* ^2^ (coefficient of determination) and *P* values (Supplementary Figs S11–S18 in Supplementary Material 5). A strong correlation between ptDNA and mtDNA would be indicated for a given parameter with *R* ^2^ close to 1 and *P*<0.05.

## Results

### DNA damage

We previously reported a decline in both the structural integrity and copy number of orgDNA molecules during development of maize and proposed that unrepaired damage led to the degradation of the orgDNAs (Oldenburg and Bendich, 2004*a*; Oldenburg *et al.*, 2006, 2013). To test this hypothesis, we employed a long-PCR procedure previously developed to assess orgDNA damage caused by genotoxic agents and/or mutations (Yakes and Van Houten, 1997; Parent *et al.*, 2011). We quantified orgDNA damage during seedling development in wild-type maize without the addition of DNA-damaging agents.

The long-PCR assay was originally used to assess DNA damage caused by a specific stressor, such as H_2_O_2_ or UV light, and reported as DNA lesions per 10kb. This method is based on the amplification of a long DNA fragment (3–20kb) and the inability of *Taq* DNA polymerase to proceed past DNA lesions such as pyrimidine dimers and oxidized pyrimidines. However, other structural features, such as single-strand gaps and double-strand breaks, would also prevent amplification of long DNA segments. Thus, the orgDNA ‘damage’ evaluated in this study may be more accurately termed DNA impediments, meaning any feature that prevents PCR amplification. We define two categories of impediment: lesions (base alterations) and discontinuities (sugar–phosphate alterations), and provide examples in Table 1. We showed here that the abundance of orgDNA impediments varied during development and among growth conditions. For maize, like other grasses, there is a developmental gradient from the base to the leaf tip (Sylvester *et al.*, 1990; Stern *et al.*, 2004). Thus, we evaluated orgDNA from four stages of development: the base of the stalk (S1), top of the stalk (S2), leaf 1 (L1), and leaf 2 (L2). The leaf number indicates the order in which maize leaves emerge; L1 emerges before L2, and thus is older. In addition, orgDNA was assessed from seedlings grown under three conditions: light-grown, dark-to-light-grown and dark-grown seedlings. Plants grown under these conditions permitted the analysis of proplastid-to-chloroplast, etioplast-to-chloroplast and proplastid-to-etioplast transitions, as well as the effect of light on mtDNA.

We performed long-PCR using ttDNA and primers to amplify a 11 207bp region of ptDNA and a 11 164bp region of mtDNA (see Materials and methods, and Supplementary Figs S1–S9 in Supplementary Material 1 at *JXB* online). The long-PCR conditions were optimized to achieve ~100% efficiency, a requirement for accurate quantification (Supplementary Figs S1–S7). A representative gel image of the long-PCR assay is shown in Supplementary Fig. S9 for mtDNA. The sensitivity of our long-PCR assay to DNA damage was confirmed by a control experiment showing that UV-light treatment of DNA inhibited amplification, leading to a faint band on the agarose gel (Supplementary Fig. S8).

**Fig. 1.**
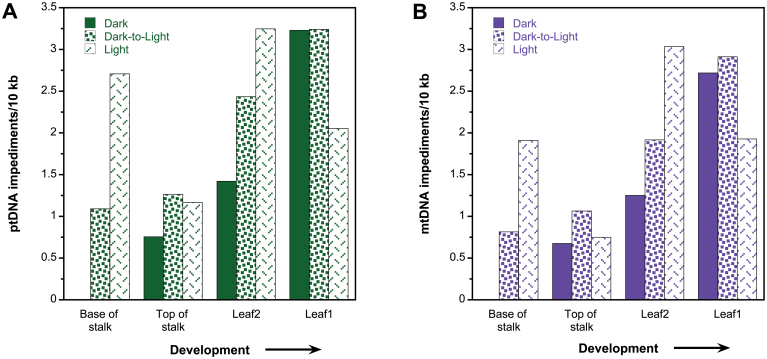
Organellar DNA damage during development of maize seedlings. Seedlings were grown under dark, dark-to-light, and light conditions, and ttDNA was prepared from the base of stalk, top of stalk, leaf 2 and leaf 1. Organellar DNA impediments per 10kb for ptDNA (A) and for mtDNA (B) were determined by amplifying ~11kb of organellar DNA using long-PCR. The relative amplification values for five biological replicates were averaged and the number of impediments determined (see Materials and methods). Impediments per 10kb are relative to the tissue with the largest amount of long-PCR amplification and were set at 0; in this case, it was the base of stalk grown in dark for both organellar DNAs.

The probability of *Taq* DNA polymerase encountering a DNA impediment is greater for long DNA (~11kb) than for shorter sections (~150bp). Thus, to measure orgDNA damage, the amount of long-PCR product was normalized to orgDNA copies measured by qPCR (see below, and Materials and methods). The tissue with the largest amount of amplified long-PCR product (and thus the least-damaged orgDNA) was used as the baseline for comparison with other tissues. This was the base of the stalk from dark-grown maize seedlings, and the values for impediments in both ptDNA and mtDNA from other tissues are given relative to this tissue (Fig. 1).

For light-grown seedlings, similar amounts of ptDNA damage (2–3 impediments per 10kb) were found in all tissues from the S1 to L1 blade, except for S2 (1 per 10kb) (Fig. 1A). In contrast, the ptDNA impediments per 10kb increased in dark and dark-to-light tissues during development (0–3 per 10kb), with the highest levels of damage in L1. Since L1 is the oldest tissue, the high level of damage for this tissue in dark-grown seedlings may reflect the fragmentation of ptDNA associated with proplastid-to-etioplast differentiation. The difference in number of impediments between dark and dark-to-light for the developing tissues (stalk tissues and L2) indicated light-induced ptDNA damage following etioplast-to-chloroplast differentiation. Overall, there was less damage in dark-grown (5.4 impediments per 10kb) than dark-to-light-grown (8 per 10kb) and light-grown (9.2 per 10kb) seedlings.

We found a similar trend for impediments per 10kb in mtDNA as was observed for ptDNA. As with ptDNA, there was a similar amount of mtDNA damage among tissues for light-grown seedlings (2 per 10kb), except for S2 (1 per 10kb). A substantial increase was observed in mtDNA damage during development for dark and dark-to-light conditions (Fig. 1B). The highest level of mtDNA damage in the dark was in L1, as was found for ptDNA. Similar to ptDNA, more mtDNA damage was found upon transfer from dark-to-light for stalk and L2, suggesting that mtDNA damage is affected by light. In addition, dark-grown and dark-to-light L1 had more mtDNA impediments than light-grown L1, probably due to higher mtDNA-damaging respiratory activity in dark-grown tissue. Although oxidative damage may occur in developing tissues (S2 and L2), the damage may be repaired, whereas in older tissue (L1) damage may persist. As with ptDNA, overall there was less damage in dark-grown (~4.7 impediments per 10kb) than in dark-to-light-grown (~6.7 per 10kb) and light-grown (~7.6 per 10kb) seedlings.

Since similar trends in DNA damage were observed for ptDNA and mtDNA, we evaluated the relationships for impediments per 10kb between the orgDNAs using regression analysis (see Materials and methods). We found a strong correlation for the amount of damage as measured by impediments per 10kb between ptDNA and mtDNA (*R* ^2^=0.97 and *P*=1.3×10^–8^; Supplementary Fig. S11). Specifically, samples with fewer impediments in ptDNA also had fewer mtDNA impediments, and those samples with higher amounts of damage in ptDNA also had more damage in mtDNA. This may indicate a common mechanism regulating DNA maintenance and repair for both organelles.

Except for L1, ptDNA and mtDNA damage overall was lower in dark-grown maize seedlings than in light-grown and dark-to-light-grown tissues. The higher damage levels in light-grown tissues and increase upon transfer from dark to light suggested that orgDNA maintenance is influenced by responses to light signals.

### orgDNA without structural impediments

**Fig. 2.**
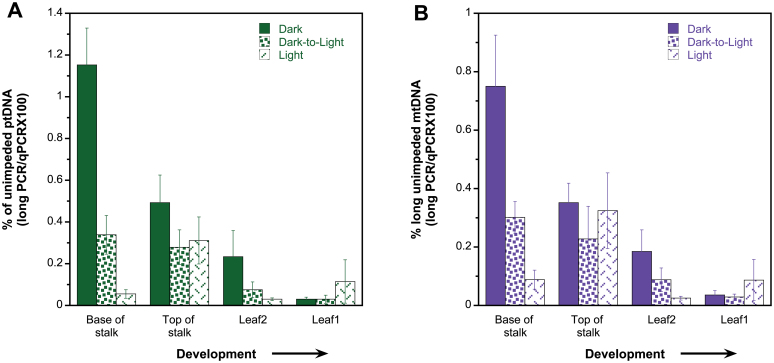
ptDNA and mtDNA without structural impediment. Seedlings were grown under dark, dark-to-light, and light conditions, and ttDNA was prepared from the base of stalk, top of stalk, leaf 2 and leaf 1 (four stages of development). DNA without impediments was determined for ptDNA (A) and mtDNA (B). Total unimpeded orgDNA copies were determined as long-PCR copies, and the percentage is given relative to orgDNA copies as determined by qPCR (see Materials and methods). Statistical analyses (ANOVA and Tukey’s HSD) were performed to compare differences between growth conditions and between developmental stages (tissues). In addition, individual sample comparisons were performed between growth conditions for each tissue and between the tissues for each growth condition. For growth conditions, dark compared with light was *P*<0.05 for both orgDNAs. For developmental stages, S1:L2, S1:L1, S2:L2, and S2:L1 was *P*<0.05 for both orgDNAs; L2:L1 was *P*<0.5 for ptDNA only. For individual sample comparisons and growth conditions, *P*<0.05 was determined for: S1 grown in dark and dark-to-light; S1 grown in dark and light; and L2 grown in dark and light (ptDNA only) for both orgDNAs. For individual sample comparisons and developmental stages, *P*<0.05 was determined for: dark-grown S1:S2, S1:L2, S1:L1, S2:L1, and L2:L1 (mtDNA only); dark-to-light-grown S1:L2, S1:L1, S2:L2 (ptDNA only), and S2:L1 (ptDNA only); and light-grown S1:L2 (ptDNA only), S2:L2, and S2:L1 for both orgDNAs. All significant comparisons are also provided in Tables S2–S6 in Supplementary Material 4. Error bars represent standard error determined for five biological replicates.

The DNA damage assay measured the relative amount of DNA impediments that inhibited *Taq* DNA polymerase amplification of long DNA sections. We also quantified ptDNA and mtDNA copies without impediments by determining the amount of DNA amplified by long-PCR. We term the DNA amplified by long-PCR as ‘unimpeded’ DNA. The amount of unimpeded orgDNA relative to total orgDNA copies (as determined by qPCR) indicated the fraction of orgDNA without any structural impediment, including DNA lesions and discontinuities (Fig. 2, Table 1). Remarkably, we found that unimpeded DNA accounted for only ~0.1–1% of all ptDNA (Fig. 2A) and mtDNA (Fig. 2B).

For ptDNA, there were significantly more unimpeded copies in dark-grown entire seedlings compared with light-grown seedlings (Fig. 2A, Supplementary Table S3). The differences in amount of unimpeded ptDNA between growth conditions may be attributed to increased oxidative damage caused by increased ROS production during photosynthesis in light and may indicate less DNA damage or more *in vivo* repair of damaged DNA in dark compared with light-exposed tissues (repair is addressed below). The light-grown seedlings contained a low amount of unimpeded ptDNA throughout development (Fig. 2A). There was, however, a 6-fold increase from S1 to S2, followed by a decline in L2 and L1 (10- and 3-fold, respectively). There were more copies in the stalk tissues (S1 and S2) than in leaf tissues (L1 and L2), and L2 had more copies than L1 (Fig. 2A, Supplementary Tables S4 and S6). We also found a decline during development in unimpeded ptDNA (10- to 37-fold) for dark and dark-to-light tissues. At the base of the stalk, we found 3- and 21-fold more unimpeded ptDNA in dark compared with dark-to-light and light conditions, respectively (Fig. 2A). For L1 of dark-grown seedlings, the amount of unimpeded ptDNA decreased with development compared with the amount found in L1 of dark-to-light tissue. This result and the large amount of damaged ptDNA reported above suggest degradation of ptDNA during proplastid-to-etioplast differentiation in the oldest dark-grown leaf, whereas light-induced damage to ptDNA was evidenced by the decrease in unimpeded ptDNA of S1 upon transfer from dark to light.

A similar trend was found for mtDNA as for ptDNA, with higher amounts of unimpeded mtDNA for dark-grown seedlings than for light-grown seedlings (Fig. 2B, Supplementary Table S3). In addition, the percentage of unimpeded mtDNA decreased during development for all growth conditions, with stalk (S1 and S2) containing more copies than L1 and L2 (Fig. 2B, Supplementary Tables S4 and S6). For light-grown tissues, we observed a 4-fold increase from the base to the top of the stalk, followed by a 13-fold decline from S2 to L2, similar to ptDNA. We found a decline from S1 to L1 for dark-to-light-grown (10-fold) and dark-grown (21-fold) tissues during development (Fig. 2B). Dark-grown S1 contained significantly higher copy numbers compared with dark-to-light and light conditions (Fig. 2B, Supplementary Table S5); however, there were similarly low levels in L1 for all three light regimes (Fig. 2B). Interestingly, light signalling appeared to influence the amount of unimpeded mtDNA, as indicated by the substantially more copies in dark conditions. Light might prompt the abandonment of repair of oxidative damage in mtDNA, as ATP generation from photophosphorylation replaces that from respiration. Finally, the decline in unimpeded mtDNA for dark-grown L1 may be attributed to the lack of repair of mtDNA damaged by ROS during respiration. As with ptDNA, changes in structure/integrity probably affect the level of unimpeded mtDNA.

The developmental changes we observed were similar for ptDNA and mtDNA: a decrease in unimpeded DNA accompanied by increasing DNA impediments. Regression analysis revealed a strong correlation for the percentage of unimpeded DNA in plastids and mitochondria (Supplementary Fig. S12 at *JXB* online), suggesting that common mechanisms influence the molecular integrity of these two orgDNAs.

In summary, dark-grown seedlings contained more orgDNA without impediments than did the light-grown seedlings. During development, the amount of orgDNA damage increased and unimpeded orgDNA decreased.

### qPCR and miPCR

qPCR is a standard method used to determine genome copy number. We previously used qPCR to assess changes in copy number for ptDNA and mtDNA during development and in response to light for maize and other plants (Oldenburg *et al.*, 2006, 2013; Shaver *et al.*, 2008; Rowan *et al.*, 2009; Zheng *et al.*, 2011). In some but not all cases, there were discrepancies between qPCR and the other methods used to assess orgDNA levels, such as a decline in ptDNA with development. We suggested that one factor contributing to the observed differences could be amplification of nuclear plastid DNA sequences (NUPTs) and nuclear mitochondrial DNA sequences (NUMTs) by orgDNA primers (Kumar and Bendich, 2011; Zheng *et al.*, 2011; Oldenburg *et al.*, 2013). Thus, we designed a method to identify qPCR primer sets that would only amplify authentic orgDNA (Kumar and Bendich, 2011) and used these primers to assess changes in maize orgDNAs. We also developed the miPCR technique, which is based on long-PCR, to assess the molecular integrity of orgDNA.

**Fig. 3.**
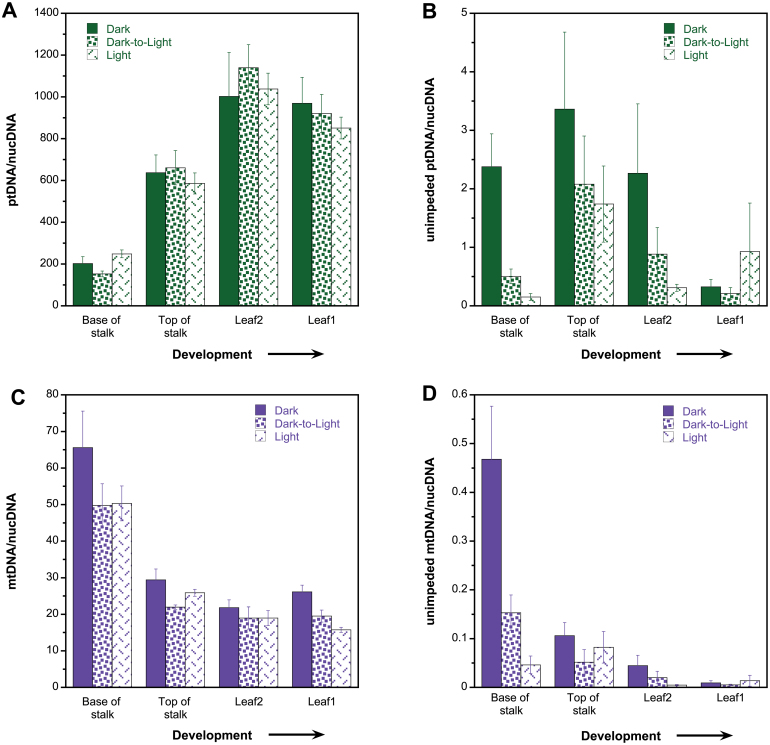
Plastid and mitochondrial genome copy number determined by qPCR and miPCR. Seedlings were grown under dark, dark-to-light, and light conditions and ttDNA was prepared from the base of stalk, top of stalk, leaf 2 and leaf 1 (four stages of development). Organellar genome copies per haploid nuclear genome were determined for ptDNA (A, B) and mtDNA (C, D). Real-time qPCR copies (A, C) were determined by normalizing short orgDNA copies with nuclear DNA copies. miPCR copies (B, D) were determined by normalizing long-PCR orgDNA copies with qPCR-determined nuclear DNA copies (see Materials and methods, and Table S1). The nuclear ploidy level was essentially constant at 2.8 during these stages of seedling development (Oldenburg *et al.*, 2006). qPCR scored most of the orgDNA copies, while miPCR scores only orgDNA copies without discontinuities or DNA lesions. Statistical analyses (ANOVA and Tukey’s HSD) were performed to compare differences between growth conditions and between developmental stages (tissues). In addition, individual sample comparisons were performed between growth conditions for each tissue and between the tissues for each growth condition. qPCR-determined copy differences were not significant for growth conditions for both orgDNAs. The developmental stages S1:S2, S1:L2, S1:L1, S2:L2, and S2: L1 had a value of *P*<0.05 for both orgDNAs. qPCR-determined copy differences were not significant for individual sample comparisons and growth conditions for ptDNA; for mtDNA, S2 grown in dark and dark-to-light, L1 grown in dark and dark-to-light, and L1 grown in dark and light conditions had a value of *P*<0.05. For individual sample comparisons and developmental stage, dark-grown S1:S2, S1:L2, and S1:L1, dark-to-light-grown S1:S2, S1:L2, and S1:L1, and light-grown S1:S2, S1:L2, S1:L1, S2:L2 (ptDNA only) and S2:L1 (mtDNA only) had *P*<0.05 for both orgDNAs. miPCR-determined copy differences were significant between dark and dark-to-light (not for mtDNA) and dark and light conditions for both orgDNAs, when growth conditions were compared. The developmental stages S1:S2 (ptDNA only), S1:L2 (mtDNA only), S1:L1(mtDNA only), S2:L2 (mtDNA only), and S2:L1. L2:L1(ptDNA only) had *P*<0.05 for both orgDNAs. For individual sample comparisons and growth conditions, only S1 grown in dark and light had *P*<0.05 for both orgDNAs. For individual sample comparisons and developmental stages, dark-grown S2:L1, dark-to-light-grown S2:L1, and light-grown S1:S2 had *P*<0.05 for ptDNA, and for mtDNA, dark-grown S1:S2, S1:L2, S1:L1, and S2:L1, dark-to-light grown S1:L2 and S1:L1; and light-grown S2:L2 and S2:L1 had *P*<0.05. All significant comparisons are also provided in Supplementary Tables S3–S6. Error bars represent standard errors determined for five biological replicates.

The dark-, dark-to-light-, and light-grown ttDNA samples were used for qPCR, and the amount of orgDNA per haploid nuclear genome (nucDNA) was quantified. For both ptDNA/nucDNA and mtDNA/nucDNA, there was no difference in copy number among the three growth conditions (Fig. 3A, C, Supplementary Table S3). For ptDNA/nucDNA, copy number increased ~5-fold during development from S1 to L1 for dark-, dark-to-light-, and light-grown tissues (Fig. 3A, Supplementary Table S6). In contrast, mtDNA/nucDNA decreased ~3-fold during development for all three growth conditions (Fig. 3C, Supplementary Tables S4 and S6). Overall, we found an increase in ptDNA/nucDNA during development, whereas mtDNA/nucDNA decreased during development.

Since we observed opposite trends between ptDNA/nucDNA and mtDNA/nucDNA, a linear regression analysis was performed. Indeed, we found a strong correlation between the orgDNAs, which was reflected by *R* ^2^ of 0.8 and *P*=0.00014 (Supplementary Fig. S13 at *JXB* online). This result suggests a common mechanism for sensing (measuring) and altering orgDNA copies but leading to different responses in plastids and mitochondria. For example, during development we found an increase in ptDNA but a reduction in mtDNA copies.

The qPCR assay measures all orgDNA amplified by the primers (usually a 100–200bp region of DNA) regardless of functional potential. For example, a *psbA* gene fragment of 150bp can be scored by qPCR even though it cannot produce a functional gene product. Moreover, molecular integrity is important not only for transcription of a single gene but for multiple genes, given the polycistronic nature of transcripts in organelles. Thus, we developed the miPCR assay to assess the molecular integrity of orgDNA. For miPCR, the unimpeded orgDNA copies generated by long-PCR were normalized to a single-copy nuclear gene (see Materials and methods; Supplementary Table S1). The most remarkable result from the miPCR assay was the very low number of unimpeded orgDNA/nucDNA copies compared with the standard qPCR assay (Fig. 3B, D). Another interesting observation was that the general trends (increase, decrease, or no change) with development and/or light conditions, as assessed by qPCR and miPCR, were similar for mtDNA but not for ptDNA.

Using miPCR, we found that dark-grown seedlings contained significantly more unimpeded ptDNA copies (average of 8.3) than dark-to-light and light-grown seedlings (average of 3.7 and 3.1 copies, respectively) (Fig. 3B, Supplementary Table S3). For example, dark-grown S1 tissue contained 5-fold and 16-fold more unimpeded ptDNA than dark-to-light-grown and light-grown stalk tissues, respectively, probably due to more DNA damage in the light. During development, the copy number of ptDNA first increased from the base to the top of the stalk (S1 to S2) and then decreased in L1 for all growth conditions (Fig. 3B, Supplementary Table S4). Although the trend was similar for all the growth conditions, there were differences in the magnitude of increase and decrease in unimpeded ptDNA/nucDNA. For example, a small increase (1.4- and 4-fold for S1 to S2) was followed by a larger decline (10- and 10-fold for S2 to L1) in dark-grown and dark-to-light-grown tissues, respectively (Fig. 3B). For the same developmental stages in light-grown tissues, however, a large increase (~12-fold) was followed by a small decrease (~2-fold).

Using miPCR, we found that dark-grown seedlings contained significantly more unimpeded mtDNA copies than light-grown seedlings (Fig. 3D, Supplementary Table S3). Furthermore, the amount of unimpeded mtDNA declined continuously from S1 to L1 (Fig. 3D, Supplementary Table S4). This same trend was also found using qPCR (Fig. 3C, Supplementary Table S4). The magnitude of decline in unimpeded mtDNA was strongly dependent on light. For example, the transition from S1 to L1 was accompanied by a decline of 50-fold for dark-grown, 29-fold for dark-to-light-grown, and only 3-fold for light-grown seedlings (Fig. 3D).

Since the patterns of change during development differed between ptDNA and mtDNA using miPCR-determined orgDNA copies, we did not expect to find a relationship when comparison was performed with linear regression analysis. This was indeed the case as indicated by a low *R* ^2^ value of 0.2 and *P*=0.14 (Supplementary Fig. S14 at *JXB* online). There were, however, enormous differences between the orgDNA/nucDNA copy number when measured by miPCR and by qPCR. We found 0.2–4 copies of unimpeded ptDNA/nucDNA using miPCR, whereas qPCR showed 200–1200 copies of ptDNA/nucDNA. The paucity of unimpeded ptDNA copies may be puzzling, especially for stalk tissue where high DNA levels would be needed for chloroplast biogenesis. This conundrum may be resolved, however, by considering the structure of replicating orgDNA and examining in-gel-prepared ptDNA with qPCR.

**Fig. 4.**
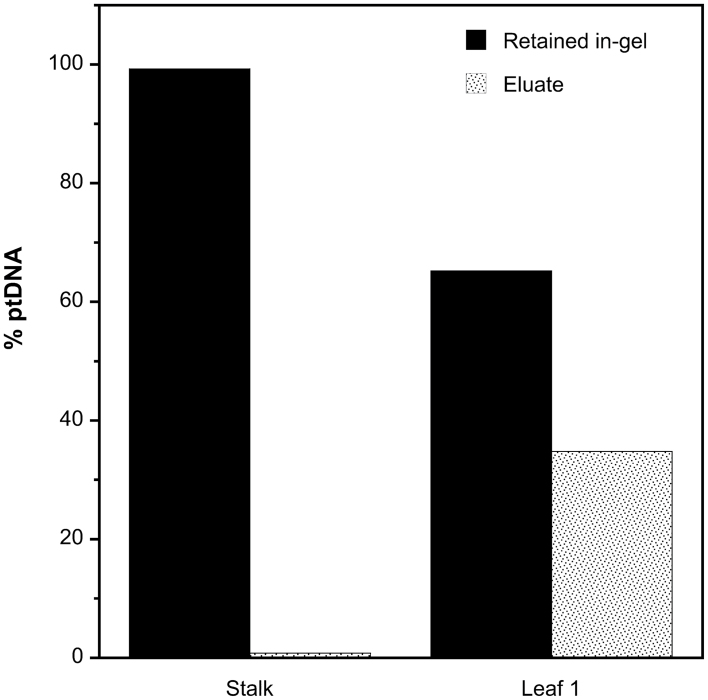
Amount of ptDNA retained in gel and eluted from the gel. Plastids were isolated from the entire stalk and leaf 1 of 14-d-old light-grown plants, then embedded in agarose, treated with lysis solution, and washed with TE buffer (see Materials and methods). The ptDNA that diffused out of the agarose was recovered from the lysis and wash solutions (eluate). The ptDNA copy number for the in-gel and eluate fractions was determined using qPCR and is given as percentage of total chloroplast DNA from both fractions. Three technical replicates were used per sample.

Plastids were isolated from the entire stalk and L1 of 14-d-old light-grown seedlings and embedded in low-melting-point agarose (see Materials and methods). The gel plugs were soaked in lysis solution and washed to yield a ‘retained in-gel’ fraction of ptDNA. The ptDNA released into the lysis solution and the wash buffer represented the diffusible fraction not retained in the gel (eluate). The in-gel and eluate fractions of ptDNA were then quantified by qPCR. For the stalk, about 99% of the ptDNA was retained in the agarose plugs, with only ~1% diffusing into the eluate (Fig. 4). Thus, most ptDNA in stalk tissue was too large to diffuse out of the gel. In contrast, nearly 35% of DNA in the green chloroplasts of L1 was present as small, diffusible fragments. These results indicated that the molecular integrity of ptDNA molecules declined greatly during maize leaf development. Our previous studies showed that ~70–90% of in-gel ptDNA from the stalk was comprised of complex, branched molecules (Oldenburg and Bendich, 2004*a*). Thus, amplification of ptDNA (and mtDNA) by long-PCR may be inhibited by discontinuities such as recombination branch points and replication forks, as well as by lesions in damaged DNA.

### DNA repair

The results from the DNA damage assay indicated that damage accumulates in both ptDNA and mtDNA during development and that the amount of damage depends on the light conditions. The basis for measuring damage is the inability of the DNA polymerase to amplify long-PCR segments. The lack of amplification may be due to impediments including DNA lesions that block *Taq* polymerase progression and discontinuities such as branching in complex forms or breakage of the DNA into fragments lacking both primer sites (Table 1). The level of damage due to lesions may be assessed using an *in vitro* assay with DNA repair enzymes.

**Fig. 5.**
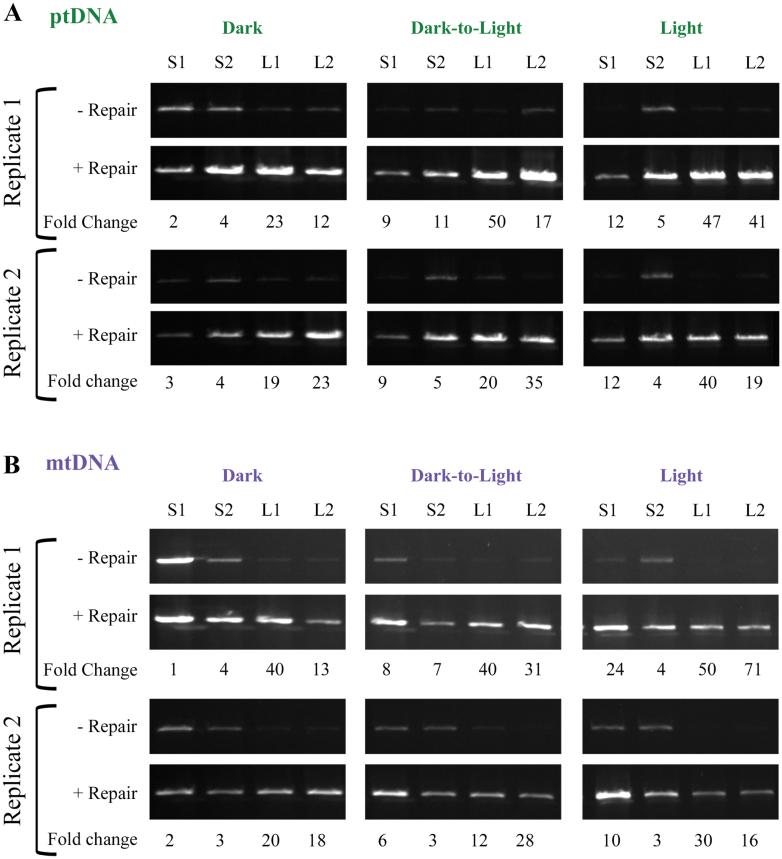
Repair of organellar DNA. Maize seedlings were grown in dark, dark-to-light and light conditions and ttDNA was prepared from base of stalk (S1), top of stalk (S2), leaf 1 (L1), and leaf 2 (L2). The amount of ~11kb long-PCR product was assessed before and after treatment with DNA repair enzymes for two biological replicates (see Materials and methods). The fold change in long-PCR product as determined by ethidium bromide-stained DNA intensity before and after repair treatment was determined and is given below the gel images. In general, there was an increase in repair from dark to dark-to-light to light conditions and during development from S1 to L1.

We performed an orgDNA repair assay on the ttDNA using a PreCR Repair kit (see Materials and methods) and long-PCR. The enzymes in this repair mix can rectify most types of DNA lesions but will not affect discontinuities except to seal a nick or fill in a short single-strand gap (Table 1). Treatment with repair enzymes resulted in an increase in the amount of long-PCR product for both ptDNA and mtDNA (Fig. 5). The magnitude of the increase was generally greater for seedlings exposed to light than grown in the dark, indicating that light may lead to orgDNA lesions. Furthermore, repair was much greater for leaf than for stalk tissues in all growth conditions, suggesting that orgDNA damage accumulates during development. The results were also consistent with the results from the damage assay: the amount of repair was directly proportional to orgDNA damage (impediments per 10kb) (Supplementary Figs S15 and S16 at *JXB* online) and inversely proportional to the percentage of unimpeded orgDNA (Supplementary Figs S17 and S18 at *JXB* online) using regression analysis.

The orgDNA may or may not be repaired with the PreCR Repair kit depending on the type of damage. For example, DNA fragmented by double-strand breaks would not be repaired, whereas oxidized bases may be repaired (Table 1). Thus, the degree of repair may be correlated with the amount of oxidatively damaged orgDNA. For example, more repair was found for ptDNA in light-grown L2 and L1 (19- to 47-fold) than S1 and S2 (4- to-12-fold) or dark-grown L2 and L1 (12- to 23-fold). These results suggest that light causes oxidative damage to orgDNA and a decline in *in vivo* repair.

## Discussion

We previously found a dramatic decrease during maize development in genome copy number for plastids and mitochondria accompanied by a change in orgDNA structure from multigenomic branched forms to linear molecules of subgenomic size (Oldenburg and Bendich, 2004*a*; Oldenburg *et al.*, 2006, 2013). We proposed that the orgDNA was damaged because of the ROS produced in these organelles and that unrepaired orgDNA was degraded. Here, we quantify orgDNA damage, copy number, and *in vitro* repair under dark, dark-to-light, and light growth conditions during seedling development.

Our results are summarized in Table 2 and revealed a strong effect of light on orgDNA damage and molecular integrity. OrgDNA from light-grown seedlings was highly damaged, with fewer unimpeded copies and more DNA repair *in vitro* than orgDNA from dark-grown seedlings, consistent with organelle-generated ROS determining the retention or degradation of orgDNA. In addition, for both ptDNA and mtDNA, we found a developmental increase in DNA impediments and a decrease in unimpeded orgDNA in both dark and dark-to-light (but not light) conditions. For ptDNA, qPCR-determined copy number increased, whereas miPCR-determined copy number first increased and then decreased. For mtDNA, copies determined by both qPCR and miPCR decreased, but the magnitude of decline was much greater with miPCR. For both orgDNAs, the number of copies per haploid nuclear genome determined by qPCR was much greater than that determined by miPCR. In order to understand the functional significance of orgDNA changes indicated by these analytical methods, we need to consider how DNA structure and integrity may affect the methods. Most of our findings apply to both ptDNA and mtDNA. For simplicity, however, the discussion below is focused on ptDNA.

**Table 2.** *Summary of orgDNA properties for seedlings grown in light, dark-to-light, and dark conditions*Dark-grown seedlings contain orgDNA with less damage and greater integrity (fewer impediments) than seedlings grown in dark-to-light and light conditions. Using the *in vitro* repair assay, orgDNA showed less repair when obtained from seedlings grown in the dark than in dark-to-light and light conditions. Using the qPCR assay, ptDNA copy number was similar among growth conditions, whereas there were more mtDNA copies in dark than in dark-to-light and light conditions. However, no difference between ptDNA and mtDNA was found when the copy number of long DNA molecules was measured using the miPCR assay. Relative levels are indicated by +, ++, and +++.

			Light	Dark-to-light	Dark
Damage		ptDNA	+++	++	+
		mtDNA	+++	++	+
% Unimpeded		ptDNA	+	++	+++
		mtDNA	+	++	+++
*In vitro* repair		ptDNA	+++	+++	+
		mtDNA	+++	+++	+
Copies	qPCR	ptDNA	+++	+++	+++
		mtDNA	+	+	+++
	miPCR	ptDNA	+	+	+++
		mtDNA	+	+	+++

### Structure and molecular integrity of orgDNA

We employed a long-PCR assay that relies on the probability of *Taq* DNA polymerase encountering an impediment in a long DNA fragment (~11kb) during amplification. The results from ptDNA damage assays under the three growth conditions (Table 2) indicated that the major cause of impediments was oxidative damage resulting from ROS produced during photosynthesis. This conclusion was supported by our finding of more *in vitro* repair, using enzymes that mend oxidative-type lesions, for light growth conditions than dark, as well as following transfer from dark to light. Some damage was evident even for dark-grown seedlings; however, this may be attributed to impediments accumulated during development. In addition to oxidative lesions, discontinuities in structure may prevent amplification of a long orgDNA fragment, as described below.

Our most remarkable result was the extremely low orgDNA/nucDNA copy number values obtained with the miPCR assay. It is difficult to reconcile such low miPCR copy number with the amount of functional orgDNA required for chloroplast or mitochondrial biogenesis. Besides DNA lesions, discontinuities such as recombination junctions, replication forks, and single-strand gaps could impede long-PCR amplification by *Taq* DNA polymerase (Table 1). In addition, some orgDNA molecules may not be long enough to serve as a template for long-PCR. Thus, discontinuities in orgDNA molecules may account for low amplification levels of long-PCR products, consistent with previous studies showing changes in orgDNA structure and integrity during development (Oldenburg and Bendich, 2004*a*; Shaver *et al.*, 2008; Rowan *et al.*, 2009).

The structure of orgDNA molecules has been assessed for maize and other plants using PFGE and DNA movies (ethidium-stained orgDNA) (Deng *et al.*, 1989; Backert *et al.*, 1995; Bendich, 1996; Shaver *et al.*, 2006). In maize, stalk tissue contains replicative forms of ptDNA comprised of branched multigenomic molecules. The low copy number of unimpeded orgDNA we now find for stalk may be attributed mainly to DNA discontinuities in such molecules, including forks and gaps previously documented for ptDNA and mtDNA (Oldenburg and Bendich, 1996; Backert *et al.*, 1997; Rowan *et al.*, 2010). We proposed that during development the replicative forms may be resolved to unit-genome-sized linear molecules and then degraded until only subgenomic linear fragments remain (Oldenburg and Bendich, 2004*a*). Here, evidence for replicative ptDNA in stalk tissue was indicated by ~99% DNA retention in the gel. In contrast, the ptDNA in the green leaf blade consisted mostly of linear molecules fragmented to less than genome size, as indicated by the large fraction (35%) of in-gel diffusible DNA (Fig. 4). Fragments shorter than 11kb would not be amplified in our long-PCR assay, contributing to the extremely low levels of unimpeded orgDNA in leaves. In some tissues, such as light-grown top of the stalk, higher levels of unimpeded ptDNA may be attributed to long-PCR amplification from unit-genome-sized molecules. In addition, we found very low levels of *in vitro* repair for the stalk (Fig. 5), suggesting little oxidative damage, as expected for non-photosynthetic tissue. Although light-grown stalk contains replicative ptDNA, PFGE shows more fragmentation to less-than-genome-sized molecules than dark-grown stalk (Oldenburg *et al.*, 2006, 2013), and our present data showed more impediments that require more *in vitro* repair in light-grown than dark-grown stalk for both orgDNAs. In general, dark-grown tissues also had more unit-genome-sized molecules that could be amplified by long-PCR than did light-grown tissues. Thus, our results using long-PCR assays were consistent with structural information revealed by PFGE and DNA movies for maize ptDNA and mtDNA.

In summary, although miPCR should report functional orgDNA, the amount will be underestimated because of developmental changes in DNA replication, molecular integrity, and damage. The underestimate would be large for meristematic cells at the base of the stalk, where long-PCR would be disrupted by the branch points associated with the recombination-dependent mode of orgDNA replication (Oldenburg and Bendich, 2004*a*; Bendich, 2010), decreasing as forks run out and replication ceases in the expanding leaf blade. Superimposed on this developmental programme is the influence of growth conditions. Photosynthesis generates ROS leading to oxidatively induced lesions and double-strand breaks. In developing chloroplasts of the stalk, ptDNA lesions may be due to ROS from the photo-oxidation of protochlorophyllide, a precursor of chlorophyll in proplastids and etioplasts (Erdei *et al.*, 2005; Solymosi and Schoefs, 2010). Development and growth conditions would also influence the levels of DNA maintenance proteins. For example, proteomic analysis of maize proplastids revealed high levels of antioxidant proteins, such as superoxide dismutase (Majeran *et al.*, 2012) that protect the ptDNA in meristematic tissues, minimizing the need for repair (Fig. 5). In addition, ROS-generating photosynthesis does not occur in proplastids, further reducing ptDNA damage and the need for repair.

Mitochondria produce most of the ROS in non-green tissues (Moller, 2001). Hence, in the dark and in meristematic tissues, one might expect more DNA damage from ROS than in light. Surprisingly, we found more unimpeded mtDNA copies in dark than light and in stalk than leaves. This may be due to DNA repair that maintains ‘good’ DNA to support respiration in dark, whereas meristematic cells at the base of the stalk probably utilize glycolysis rather than respiration (Bendich, 2010; Kelliher and Walbot, 2014) in hypoxic conditions. Similar trends for ptDNA and mtDNA concerning damage, unimpeded copies, and *in vitro* repair suggest retrograde and inter-organellar signalling (Foyer and Noctor, 2003; Wright *et al.*, 2009). Signalling from plastids to mitochondria was also suggested by more damage when dark-grown plants were transferred to light. The effect of light on mtDNA damage may also be associated with a change in mitochondrial function as photophosphorylation replaces respiration.

A strong relationship between ptDNA and mtDNA for orgDNA damage (impediments per 10kb), long-PCR (% unimpeded orgDNA), and copies determined by qPCR (Supplementary Figs S11–S13) also indicates a common mechanism governing DNA maintenance and repair. Common repair pathways may include DNA repair enzymes targeted to both organelles, such as RECA2 in *Arabidopsis* (Shedge *et al.*, 2007). Thus, we may expect similar dual-targeted proteins maintaining copy numbers in both organelles (Carrie and Small, 2013).

**Fig. 6.**
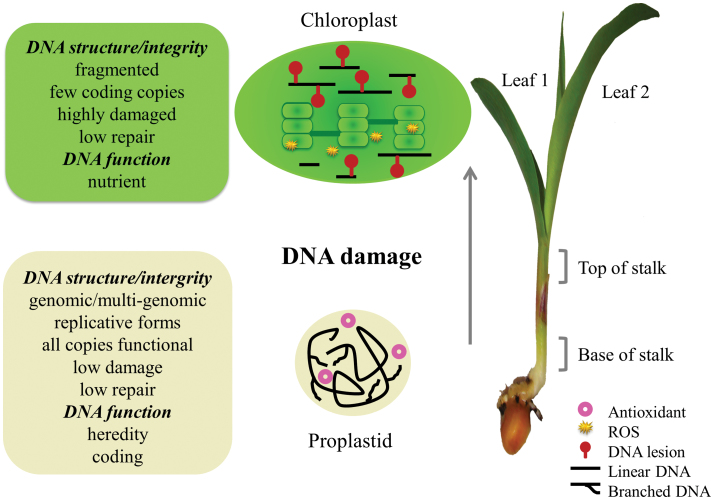
Changes in orgDNA during maize development. Recombination-dependent replication of orgDNA in the basal meristem produces branched, multigenomic chromosomes in proplastids and mitochondria (not depicted). DNA-damaging oxidative stress is minimized, requiring little repair, by maintaining hypoxia, antioxidants, and no ROS-generating photosynthesis or respiration. Early in leaf development, orgDNA damage occurs due to ROS generated in photosynthesis, respiration, and oxidation of pigments and lipids. Later, when the damage level exceeds the repair capacity, orgDNA is fragmented and no longer functions in coding or heredity, mitochondria switch from respiration to photorespiration, and DNA copy number declines faster for mitochondria than for chloroplasts.

The changes in DNA that accompany maize seedling development seem complex. These changes are summarized in Fig. 6.

### Functional organellar DNA and methods for orgDNA quantification

How can PCR-based methods designed for copy number and damage analysis of long DNA molecules be used to evaluate the functional significance of highly fragmented molecules? What, if any, function is served by orgDNA fragments less than the size of a gene?

Various methods have been used to assess copy number of orgDNA in isolated organelles [PFGE, 4’,6-diamidino-2-phenylindole (DAPI)-DNA, and DNA movies] and total tissue DNA (DNA reassociation kinetics, qPCR, and restriction/blot hybridization). The former methods uniformly indicate a decline in orgDNA during development in the light, whereas the latter methods frequently show little or no change (Li *et al.*, 2006; Zoschke *et al.*, 2007; Rowan and Bendich, 2009; Oldenburg *et al.*, 2014). Previously, we proposed three factors that contributed to the discrepancy: (i) ignoring the fact that leaf tissue is comprised of different cell types and not all cells would contain equal amounts of orgDNA (Rowan and Bendich, 2009; Zheng *et al.*, 2011); (ii) the age of plant tissues used for comparison (Rowan and Bendich, 2009); and (iii) interference due to NUPTs/NUMTs (Kumar and Bendich, 2011). Here, although we used orgDNA-specific primers to avoid NUPTs/NUMTs, we still found the same disagreement between qPCR and other methods. With qPCR, the copy number of ptDNA/nucDNA actually increased during development for light-grown seedlings (Fig. 3A), in contrast to other methods that indicated a decrease. Our miPCR results also showed a decrease and provided an explanation for the discrepancy among methods: the potential to overestimate orgDNA copy number by qPCR and restriction/blot hybridization.

Standard qPCR measures essentially all orgDNA in ttDNA regardless of size and molecular integrity since only a short DNA segment (100–200bp) is amplified. Thus, for tissues with highly fragmented orgDNAs, such as light-grown L1, qPCR and restriction/blot hybridization could lead to an overestimate of *functional* genome copies. For tissues with multigenomic forms, however, qPCR should give an accurate estimate of functional orgDNA. In contrast, miPCR measures only orgDNA >11kb, which would include both multigenomic complexes and genomic linear molecules. All of the long-PCR-determined copies (from miPCR) are free of impediments and are, therefore, functional; such copies decline with development and in response to light, reflecting the decline revealed by DAPI-DNA, PFGE, and DNA movies. Much of the orgDNA from maize stalk (and cultured tobacco and liverwort cells) remains immobile after PFGE, and low-resolution DNA movies revealed large blobs of ethidium bromide staining from which long and branched fibres emanate (Oldenburg and Bendich, 1996, 1998, 2004*b*). These complex structures were interpreted as branched replicating orgDNA. High-resolution electron microscopy of analogous mtDNA structures from cultured *Chenopodium album* cells revealed a high density of multiply branched DNA, also interpreted as replicating mtDNA (Backert and Börner, 2000). Such replicating forms would contain closely spaced impediments, leading to an underestimate of functional orgDNA in the miPCR assay.

The qPCR assay is suitable to quantify DNA where all the templates are uniform. But for maize orgDNA extracted from green L1, a 300-bp fragment that could not encode a typical protein would nevertheless be scored by qPCR. Given the polycistronic nature of organellar transcripts (Liere *et al.*, 2011; Zhelyazkova *et al.*, 2012), what function could be served by the transcription of (presumably highly fragmented) ptDNA reported for green L1 tissue of barley (Emanuel *et al.*, 2004)? Non-coding RNAs involved in post-transcriptional gene regulation is one possibility (Hotto *et al.*, 2012). Another involves the monitoring of DNA damage. Transcription-coupled repair is one of many processes by which DNA damage is repaired in bacteria and the nucleus (Deaconescu *et al.*, 2012), and transcription has been proposed as a global surveyor of DNA damage (Epshtein *et al.*, 2014). Transcription-coupled repair may be expected to operate in plastids and mitochondria early in development of these organelles. As the damage load accumulates in developing leaves, transcription may continue even though orgDNA has been ‘abandoned’ because repair proteins are no longer supplied from nucleus-encoded genes (RecA, for example). In this scenario, the residual transcription of highly fragmented orgDNA superficially suggests a coding function for such fragments. Instead, we suggest that such transcripts do not benefit the cell because of their coding or structural potential and inaccurately inflate the copy number estimate of functional organellar genomes.

Whereas quantification of mitochondrial RNA and DNA (by qPCR) has been reported in other plants (Li *et al.*, 2006; Preuten *et al.*, 2010), parallel data for mtDNA molecular integrity have not been reported. Nonetheless, Liere *et al.* (2011) stated that ‘No correlation between gene copy numbers and transcript levels were found during leaf development in Arabidopsis (Preuten *et al.*, 2010) and *Phaseolus vulgaris* (Woloszynska and Trojanowski, 2009)’, consistent with an overestimation of functional mtDNA copy number by qPCR in mature leaf tissue.

### Concluding remarks

At early stages of organellar development, fully functional orgDNA is needed for subsequent respiration and photosynthesis, just as fully functional nuclear DNA is needed, but copy number changes only for orgDNA. As leaves develop and the physiological roles of the organelles change, the maintenance of high-copy-number orgDNA (but not nuclear DNA) lessens, damage persists, and orgDNA is degraded. This abandonment of orgDNA is abrupt for maize and gradual for ptDNA in some dicot plants (Shaver *et al.*, 2006; Rowan and Bendich, 2009; Oldenburg *et al.*, 2014). Energy metabolism poses a much greater threat to the genomes in mitochondria and chloroplasts than in the nucleus. Whereas DNA repair suffices for the nucleus, orgDNA turnover, copy number change, and abandonment are also needed to maintain cellular homeostasis during development.

## Supplementary Data

Supplementary data are available at *JXB* online.

Supplementary Material 1. The use of long-PCR to quantify organellar DNA copy number and to assess organellar DNA damage.

Supplementary Fig. S1. PCR products fractionated on agarose gels for ptDNA (A) and for mtDNA (B).

Supplementary Fig. S2. PCR products fractionated on agarose gels for ptDNA (A) and for mtDNA (B).

Supplementary Fig. S3. PCR products fractionated on an agarose gel for ptDNA.

Supplementary Fig. S4. PCR product band intensities determined with ImageJ (Schneider *et al.*, 2012) over 16 to 21 cycles for ptDNA with 7.5ng and 15ng total tissue DNA.

Supplementary Fig. S5. PCR products fractionated on agarose gels for ptDNA (A) and for mtDNA (B).

Supplementary Fig. S6. PCR products over three PCR cycles in the exponential phase for ptDNA (blue) and mtDNA (red).

Supplementary Fig. S7. A standard curve generated by fractionating MassRuler ladders with different concentrations (in ng) and band intensities (in pixels) for a 10kb band.

Supplementary Fig. S8. PCR products of ttDNA treated with or without UV fractionated by agarose gel electrophoresis.

Supplementary Fig. S9. Controls and long-PCR products fractionated by agarose gel electrophoresis.

Supplementary Material 2.

Supplementary Table S1. DNA copies determined using real-time qPCR, long-PCR and miPCR.

Supplementary Material 3. Validation of PreCR Repair kit.

Supplementary Fig. S10. Agarose gel electrophoresis of lambda DNA either not digested or digested with *Xmn*I (A). PCR products fractionated on agarose gels of UV-treated or *Xmn*I digested DNA (B).

Supplementary Material 4. Statistical analysis.

Supplementary Table S2. Abbreviations.

Supplementary Table S3. Tukey comparisons of growth conditions.

Supplementary Table S4. Tukey comparisons of developmental stages.

Supplementary Table S5. Tukey multiple comparisons of growth conditions for each tissue.

Supplementary Tables S6. Tukey multiple comparisons of tissues for each growth condition.

Supplementary Material 5. Regression analysis.

Supplementary Fig. S11. Correlation between ptDNA and mtDNA for impediments/10kb.

Supplementary Fig. S12. Correlation between ptDNA and mtDNA for percentage of unimpeded long orgDNA copies.

Supplementary Fig. S13. Correlation between ptDNA/nucDNA and mtDNA/nucDNA determined by qPCR.

Supplementary Fig. S14. Correlation between ptDNA and mtDNA for unimpeded orgDNA/nucDNA.

Supplementary Fig. S15. Correlation between fold ptDNA repair and ptDNA impediments/10kb.

Supplementary Fig. S16. Correlation between fold mtDNA repair and mtDNA impediments/10kb.

Supplementary Fig. S17. Correlation between fold ptDNA repair and % unimpeded ptDNA.

Supplementary Fig. S18. Correlation between fold mtDNA repair and % unimpeded mtDNA.

Supplementary Data

## Abbreviations:

ANOVA: analysis of variance
DAPI: 4’: 6-diamidino-2-phenylindole
HSD: honestly significant difference
miPCR: molecular integrity PCR
mtDNA: mitochondrial DNA
nucDNA: nuclear DNA
NUMTs: nuclear sequences of mitochondrial origin
NUPTs: nuclear sequences of plastid origin
orgDNA: organellar DNA
Pchlide: Protochlorophyllide
PFGE: pulsed-field gel electrophoresis
ptDNA: plastid DNA
qPCR: real-time quantitative PCR
ROS: reactive oxygen species
ttDNA: total tissue DNA.
