# Supplementary material for: Changes in DNA damage, molecular integrity, and copy number for plastid DNA and mitochondrial DNA during maize development
Source: J Exp Bot. 2014 Sep 26;65(22):6425–39. doi: 10.1093/jxb/eru359 (PMC4246179; doi:10.1093/jxb/eru359)
Supplement: Supplementary Data [file supp_65_22_6425__index.html]

Changes in DNA damage, molecular integrity, and copy number for plastid DNA and mitochondrial DNA during maize development — Changes in DNA damage, molecular integrity, and copy number for plastid DNA and mitochondrial DNA during maize development — Supplementary Data 

# Changes in DNA damage, molecular integrity, and copy number for plastid DNA and mitochondrial DNA during maize development

## Supplementary Data

Data files

**Files in this Data Supplement:**

- Supplementary Data - Supplementary Data
