# Supplementary material for: Changes in DNA damage, molecular integrity, and copy number for plastid DNA and mitochondrial DNA during maize development
Source: J Exp Bot. 2014 Sep 26;65(22):6425–39. doi: 10.1093/jxb/eru359 (PMC4246179; doi:10.1093/jxb/eru359)
Supplement: Supplementary Data [file supp_eru359_jexbot131920_file001.pdf]

Title: Changes in DNA damage, molecular integrity, and copy number for plastid DNA and mitochondrial DNA during maize development

Authors: Rachana A. Kumar, Delene J. Oldenburg and Arnold J. Bendich

Supplemental material 1

The use of long PCR to quantify copy number and assess damage for organellar DNA

Our objective was to use long PCR to determine the copy number of ptDNA and mtDNA in total DNA extracted from tissue samples. In previous work, the efficiency of amplification of DNA decreased as the target sequence length increased above about 1 kb, so that DNA quantification using long PCR was considered unreliable (Arezi et al. 2003). Here, we used a long-PCR end-point procedure to amplify organellar DNA fragments of ~11 kb and performed quantification using gel electrophoresis, ethidium bromide (EtBr)-DNA fluorescence, and DNA mass standards. We achieved an efficiency of ~100% and validated the method for assessing orgDNA damage. These ptDNA and mtDNA primers and long-PCR conditions were used:

nad4, 11164 bp mtDNA-specific

nad4_F3, 5'-GTTGGACCACAGGCAAAAGT-3'

trnk_R1, 5'-GCGAGGAATGGAAGCAGTAG-3'

rps14, 11207 bp ptDNA specific

rps14_F1, 5'-ATCTTGTTGCACCCGGTAAC-3'

rps14_R5, 5'-TATCCTGACCCTTTCTTGTGC-3'

Steps	Temperature (°C)	Duration
Initial Denaturation	94	30 s
Annealing	65 (ptDNA), 61 (mtDNA)	1 min
Extension	65	7 min 10s
Final extension	65	10 min
Total number of cycles: 20 for ptDNA and 22 for mtDNA		

1. **Extension time and temperature**: Using a typical extension temperature of about 70°C, we obtained very little product. We then tested 65°C and 68°C, and the amount of product increased. We also found that the duration of extension step of 39 s/kb gave the best results for both ptDNA and mtDNA (data not shown). An extension temperature of 65°C was chosen for further use for both ptDNA (Fig. S1A) and mtDNA (Fig. S1B).

A B

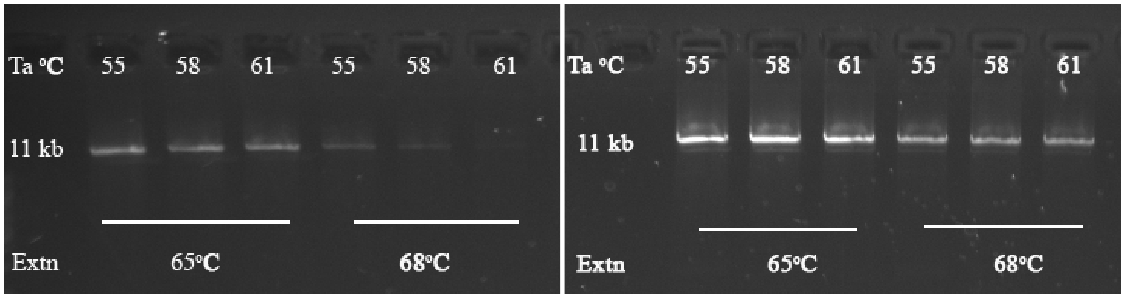

Fig. S1 PCR products fractionated on agarose gels for ptDNA (A) and for mtDNA (B).

2. **Annealing temperature and Mg^{++} concentration**: We performed PCR using a range of annealing temperature (Ta) values from 55 to 65°C. We also tested Mg^{++} concentrations from 1 to 2.2 mM at these Ta values. We chose a high Ta that did not result in decreased PCR product and a low Mg^{++} concentration that did not yield non-specific products (DNA sizes other than 11 kb). We determined that the optimal Ta was 65°C and 61°C and Mg^{++} concentration was 1.9 mM and 1.6 mM for ptDNA (Fig. S2A) and mtDNA (Fig. S2B) primers, respectively.

A

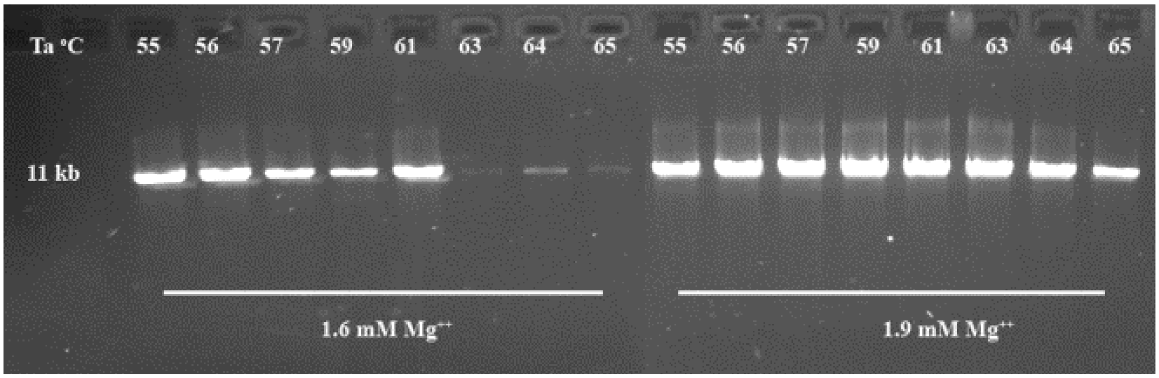

B

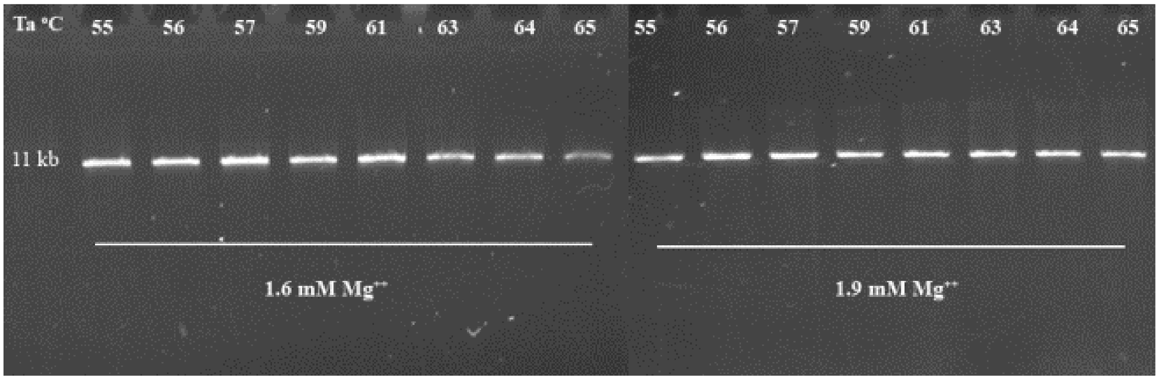

Fig. S2 PCR products fractionated on agarose gels for ptDNA (A) and for mtDNA (B).

3. **Initial denaturation time**: Fig. S3 shows that the duration of the initial denaturation (at 94°C) affected the PCR product. There was about two-fold less PCR product at 210 s then at 30s, most likely due to greater thermal scission of the DNA strands with the longer time. Thus we adopted 30 s as the initial denaturation time.

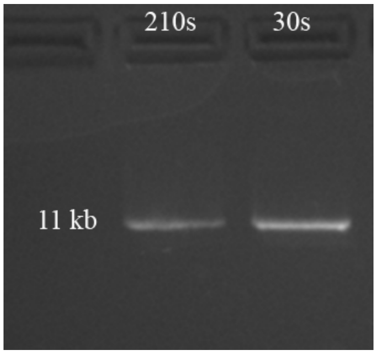

Fig. S3 PCR products fractionated on an agarose gel for ptDNA.

We optimized the Ta and chose 61° and 65°C for ptDNA and mtDNA, respectively. We also found the optimal Mg^{++} concentration to be 1.9 mM and 1.6 mM for ptDNA and mtDNA, respectively. Finally, the extension temperature of 65°C at 39s/kb and denaturation time of 30 s was found to be optimal for both ptDNA and mtDNA.

4. **DNA polymerase:** Phusion, LongAmp *Taq* Polymerase (NEB, USA) and GeneAmp XL PCR kit (GE Life Technologies, USA) were compared. We found LongAmp *Taq* to be the better of the two enzymes since it had better efficiency and higher specificity (data not shown; see below).

5. **DNA extraction method and integrity of total tissue DNA:** We tested several DNA extraction methods, including column-based, resin-based and CTAB procedures. We found that CTAB gave the highest yield of organellar DNA. After extraction, the total tissue DNA was fractionated by agarose gel electrophoresis to assess the size of the DNA. All of the DNA was found in the compression zone of the gel (where DNA sizes >50 kb accumulate), and there was no smearing or laddering below ~50 kb (Oldenburg et al. 2013).

6. **Cycle number optimization and efficiency**: We performed long PCR over a range of cycles for both ptDNA and mtDNA and determined the cycle number for the approximate mid-point in the exponential phase. For example, 20 cycles was used for ptDNA (Fig. S4 and Fig. S5A) and 22 cycles for mtDNA (Fig. S5B).

For both ptDNA and mtDNA, the EtBr-DNA fluorescence intensity was about two times greater using 15 ng of total tissue DNA compared to using 7.5 ng and only one PCR

product was produced with a size of ~11 kb, the interval between primers (Fig. S5). Thus, the long PCR procedures appear to be quantitative and in the exponential range for these amounts of input DNA. Furthermore, these two DNA amounts were included as controls (see Fig. S8) in all long-PCR experiments to validate the assay (see item 10, below) and to verify that the cycle number was in the exponential range for tissues with different levels of long unobstructed orgDNA.

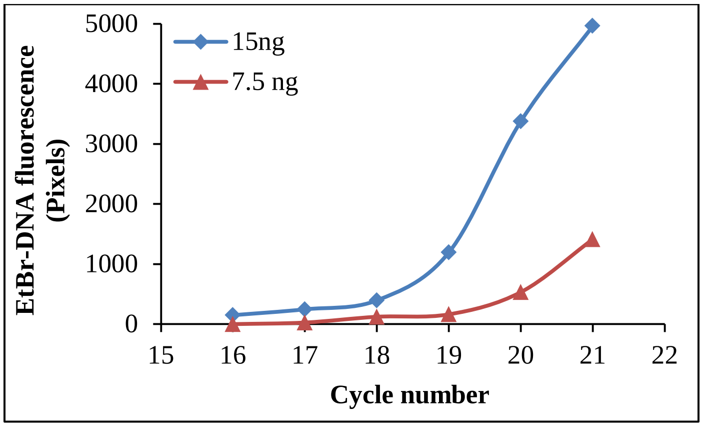

Fig. S4 PCR product band intensities determined with ImageJ (Schneider et al. 2012) over 16 to 21 cycles for ptDNA with 7.5 ng and 15 ng total tissue DNA.

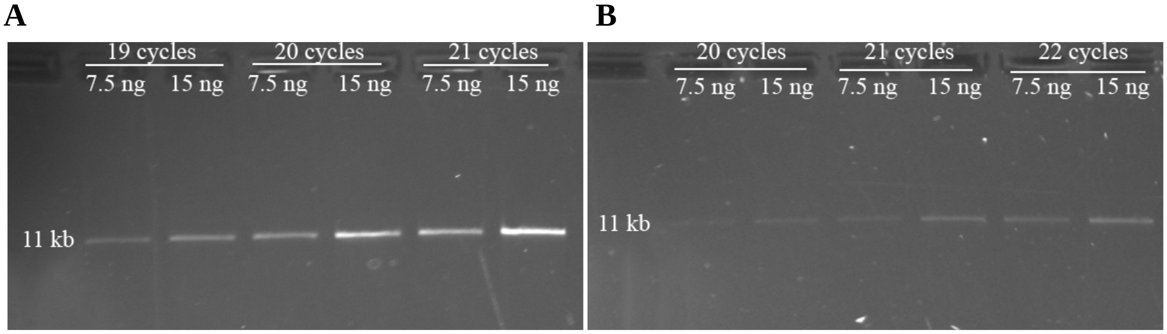

Fig. S5 PCR products fractionated on agarose gels for ptDNA (A) and for mtDNA (B).

To determine the efficiency of long PCR, we modified a method commonly used for calculating qPCR efficiency (Pfaffl 2001). We selected three points from the exponential curve and plotted the log of product-band intensity at a given cycle number. The slope of the line was then used to calculate the efficiency using the equation $10^{-(1/slope)}$ and determined as 2.1 or 110% for ptDNA and 2.0 or 100% for mtDNA (Fig. S6). Generally acceptable efficiencies for qPCR are between 90-110%.

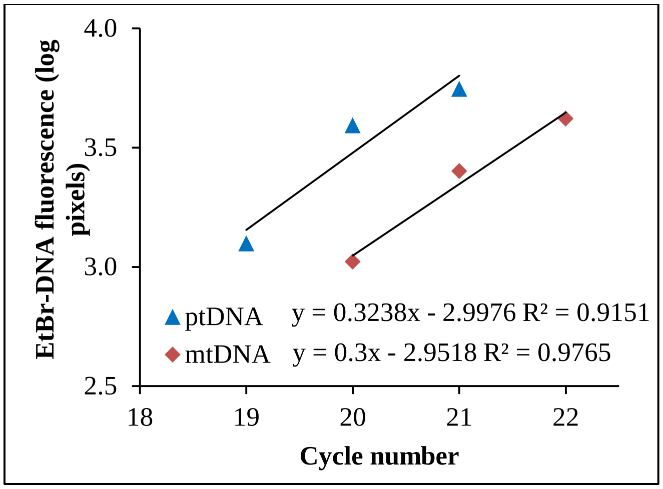

Fig. S6 PCR products over three PCR cycles in the exponential phase for ptDNA (blue) and mtDNA (red). PCR products were quantified by measuring band intensities (in pixels) on agarose gels using ImageJ.

7. **Long PCR with nuclear DNA primers:** We conducted long PCR using primers for several single-copy nuclear genes using various primers and Ta values, but always obtained multiple PCR products rather than a single product, as we obtained with ptDNA and mtDNA. The multiple agarose gel bands we observed can be attributed to the many *adh1*-like sequences interspersed among other highly repetitive sequences in the maize nuclear genome (Schnable et al. 2009). Thus we could not use a purely PCR-based method to determine copy number of organellar DNA per nuclear genome.

8. **Gel quantification of long-PCR products using DNA mass standards:** A DNA MassRuler (Fermentas) was used to quantify long-PCR products. A MassRuler standard curve (Fig. S7) was produced using a dilution series (0.8 to 38 ng load) (see Fig. S9), fractionation on an agarose gel, and measurement of the EtBr-DNA fluorescence (pixels) of the 10 kb standard (using ImageJ). DNA copy number at the end of the PCR was then calculated using the following equation:

   (Amount of DNA in ng$\times 6.022\times10^{23}$) $\div$ (size of PCR product in bp$\times10^{9}\times650$).

   Where, 6.022×10^{23} is Avogadro's number, the size of long PCR product for ptDNA is 11207 bp and 11164 bp for mtDNA, average weight of a base pair (bp) is 650 daltons, and multiplication by 10^9 for ng conversion.

   Finally, copies of orgDNA in template DNA was determined by

   Number of copies at the end of PCR $\div 2^{\text{number of cycles}}$.

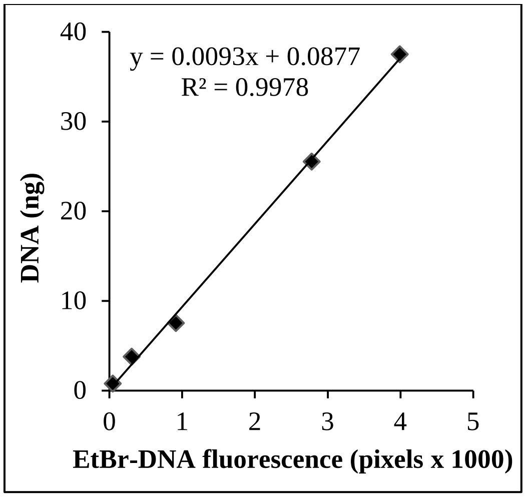

Fig. S7 A standard curve generated by fractionating MassRuler ladders with different concentrations (in ng) and band intensities (in pixels) for a 10 kb band.

9. **Comparison of qPCR-quantification and gel-quantification**: We confirmed that our gel quantification method is as sensitive as fluorophore-based DNA quantification methods, such as SYBR-G or Pico-G, by the following procedure. We used qPCR to produce the nuclear *adh1* gene product at 26 cycles (in the exponential range). This *adh1*-PCR standard was quantified using Quant-it kit (Life Technologies, NY), a fluorophore-based method. We determined the copies to be 1000. This qPCR product was then fractionated on an agarose gel and the template copies were determined to be 1178 as described above.

10. **Validation of long PCR:** ttDNA from light-grown top of stalk tissue (S2) was either treated with UV (~240 J/m^2) using Stratalink (+UV) or left untreated (-UV). A long PCR was performed to amplify an ~11 kb region of ptDNA and a short PCR was performed to amplify a 207 bp region of ptDNA. Short PCR served as a control for the amount of input DNA, which is evident by the presence of equal intensity bands in +/- UV samples. The presence of a very faint 11-kb band in +UV, compared with –UV, indicates that impediments due to UV treatment (pyrimidine dimers) can block the polymerase and decrease amplification, showing the sensitivity of the long-PCR procedure in detecting DNA lesions.

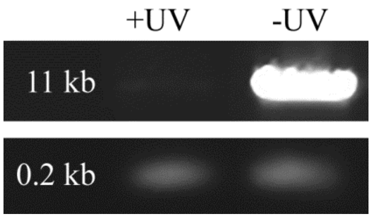

Fig 8 PCR products of ttDNA treated with or without UV fractionated by agarose gel electrophoresis.

11. **Performing long PCR on tissue samples**: Fig. S9 shows the agarose gel-fractionation of the products from a typical long PCR experiment, using the optimized conditions described above. We include several controls in every long-PCR experiment: no DNA template control (NTC), template control (ctl) with 15 ng (100%) and 7.5 ng (50%) total tissue DNA, and MassRuler DNA standards. The assay is validated only if the EtBr-DNA fluorescence intensity of the 50% ctl is between 40-60% of the 100% ctl. For example, in Fig. S9, the 50% ctl is 54% of the 100% ctl, validating the assay.

    To assess orgDNA lesions/10 kb, the sample band intensities are normalized to qPCR-determined orgDNA copies, as described in Materials and methods. The sample with highest normalized intensity is set at 1 and used to determine the relative amplification for the other samples.

    Using a standard curve generated by MassRuler (as shown in Fig S7), we determined the long-template copies for the samples as described above. These copies were then normalized to a single copy nuclear gene, *adh1*, for miPCR quantification.

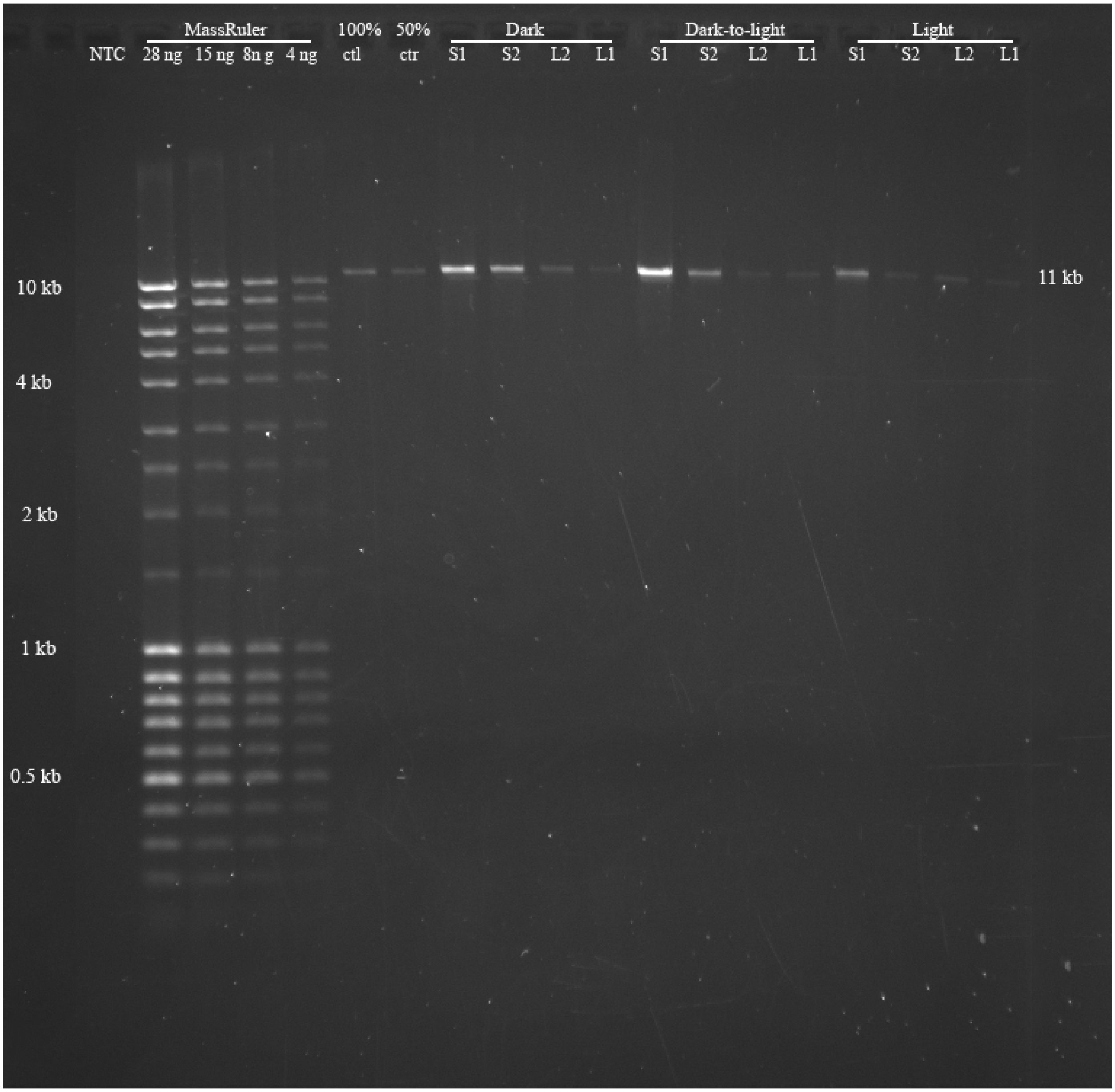

Fig. S9 Controls and long PCR products fractionated by agarose gel electrophoresis. S1 is the base of the stalk, S2 is the top of the stalk and L2 and L1 are leaf 1 and leaf 2, respectively. The 11-kb mtDNA fragment is the only PCR product for each tissue, and the copy number varies among tissues. The 100% and 50% controls were used for every long PCR reaction, and this control was prepared from light-grown stalk tissue.

Conclusions

After optimizing several variables, we were able to achieve ~100% efficiency for both ptDNA and mtDNA primers. We also established that the gel-quantification method is as sensitive and reliable as fluorophore-based methods. The previous inability to achieve ~100% efficiency for long PCR can be attributed to inhibition of *Taq* polymerase by the SYBR Green in the reaction mix. Hence, for long PCR we did not include any DNA fluorophore. Instead, we optimized the traditional end-point PCR method and measured the EtBr-stained long-PCR product to quantify orgDNA. Our long-PCR assay at ~100% efficiency can be used to quantify copy number and assess impediments in orgDNA.

References:

Arezi B, Xing W, Sorge JA, Hogrefe HH (2003) Amplification efficiency of thermostable DNA polymerases. Anal. Biochem. 321: 226-235

Oldenburg DJ, Kumar RA, Bendich AJ (2013) The amount and integrity of mtDNA in maize decline with development. Planta 237: 603-617

Pfaffl MW (2001) A new mathematical model for relative quantification in real-time RT-PCR. Nucleic Acids Res. 29: 6

Schnable PS, Ware D, Fulton RS, et al. (2009) The B73 Maize Genome: Complexity, Diversity, and Dynamics. Science 326: 1112-1115

Schneider CA, Rasband WS, Eliceiri KW (2012) NIH Image to ImageJ: 25 years of image analysis. Nat. Methods 9: 671-675

Title: Changes in DNA damage, molecular integrity, and copy number for plastid DNA and mitochondrial DNA during maize development

Authors: Rachana A. Kumar, Delene J. Oldenburg and Arnold J. Bendich

Supplemental Material 2

Table S1 DNA copies determined using real-time qPCR, long PCR, and miPCR

Data are shown for one of the five biological replicates for qPCR and long-PCR assays and used to calculate org DNA copy number. Absolute values from qPCR and long-PCR assays were determined using DNA standards (Materials and methods).

Growth Condition	Tissues	**A** nucDNA copies (qPCR)[a]	**B** ptDNA copies (qPCR)[b]	**C** ptDNA copies (long PCR)[c]	**C/B×100** % unimpeded ptDNA[d]	**B/A** ptDNA/ nucDNA (qPCR)[e]	**C/A** unimpeded ptDNA/nucDNA (miPCR)[f]
Dark	Base of stalk	10316	3156667	30453	0.96	306	2.95
	Top of stalk	12028	9096667	13952	0.15	756	1.16
	Leaf 2	22020	15995833	33458	0.21	726	1.52
	Leaf 1	22742	19361667	2080	0.01	851	0.09
Dark-to-light	Base of stalk	17563	3427500	12703	0.37	195	0.72
	Top of stalk	9033	7330833	30657	0.42	812	3.39
	Leaf 2	11601	13617500	30715	0.23	1174	2.65
	Leaf 1	16809	17706667	1899	0.01	1053	0.11
Light	Base of stalk	20816	5715000	5572	0.10	275	0.27
	Top of stalk	15252	8848333	53449	0.60	580	3.50
	Leaf 2	15618	16776667	5528	0.03	1074	0.35
	Leaf 1	21373	16005000	3122	0.02	749	0.15

Growth Condition	Tissues	**A** nucDNA copies (qPCR)[a]	**D** mtDNA copies (qPCR)[b]	**E** mtDNA copies (long PCR)[c]	**E/D×100** % unimpeded mtDNA[d]	**D/A** mtDNA/ nucDNA (qPCR)[e]	**E/A** unimpeded mtDNA/nucDNA (miPCR)[f]
Dark	Base of stalk	10316	1009667	5636	0.56	98	0.546
	Top of stalk	12028	365000	623	0.17	30	0.052
	Leaf 2	22020	532083	891	0.17	24	0.040
	Leaf 1	22742	580667	78	0.01	26	0.003
Dark-to-light	Base of stalk	17563	1167667	4136	0.35	66	0.235
	Top of stalk	9033	194600	959	0.49	22	0.106
	Leaf 2	11601	355833	826	0.23	31	0.071
	Leaf 1	16809	367833	84	0.02	22	0.005
Light	Base of stalk	20816	1051333	1574	0.15	51	0.076
	Top of stalk	15252	401833	2643	0.66	26	0.173
	Leaf 2	15618	358333	105	0.03	23	0.007
	Leaf 1	21373	372417	103	0.03	17	0.005

[a] Genome copy number of nucDNA per ng of ttDNA was determined by qPCR based on the standard curve for a single copy gene, *adh1*.
[b] Genome copy number of ptDNA or mtDNA per ng of ttDNA was determined by qPCR based on the standard curve for a single copy gene, *rps14* or *nad4*, respectively.
[c] Genome copy number of ptDNA or mtDNA per ng of ttDNA was determined by long PCR and based on a standard curve using DNA ladder standards (MassRuler).
[d] The percentage of unimpeded orgDNA was determined by dividing long orgDNA copies by qPCR-determined orgDNA copies and multiplying by 100.
[e] orgDNA copies per haploid nucDNA was determined by dividing qPCR-determined orgDNA copies by nucDNA copies.
[f] orgDNA copies per haploid nucDNA using miPCR was determined by dividing long orgDNA copies by nucDNA.

Title: Changes in DNA damage, molecular integrity, and copy number for plastid DNA and mitochondrial DNA during maize development

Authors: Rachana A. Kumar, Delene J. Oldenburg and Arnold J. Bendich

Supplemental material 3

Validation of PreCR repair kit

The PreCR repair kit (New England Biolabs, MA) was validated using the UV-treated control lambda DNA, provided by the manufacturer, as well as with the fragmented lambda DNA prepared by digesting with *Xmn*I. A complete digestion was confirmed by fractionating the products on agarose gel (Fig. S9A). UV-treated and fragmented lambda DNA was either incubated with enzyme mix (+Repair) or left unrepaired (-Repair). PCR was performed to amplify a 1-kb product using L1 primers and UV-treated and fragmented lambda DNA. The PCR products were fractionated to compare band intensities for +Repair and –Repair (Fig. S9B). The PreCR repair enzyme mix successfully repaired the UV-treated DNA but not the fragmented DNA.

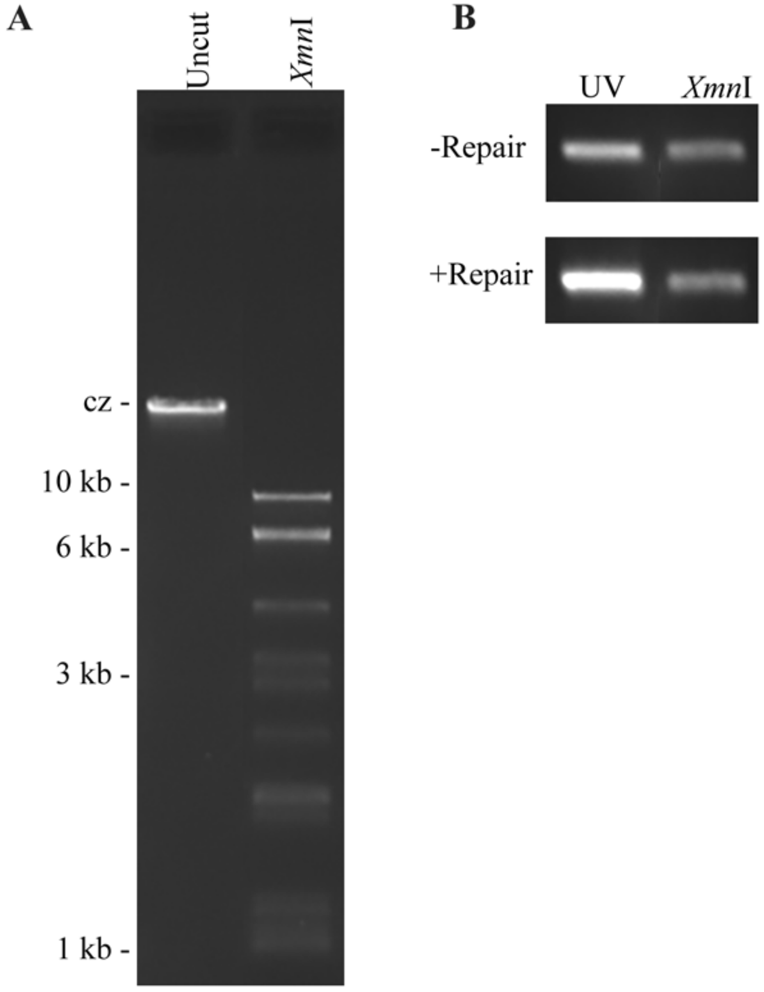

Fig. S10 Agarose gel electrophoresis of lambda DNA either not digested or digested with *Xmn*I (A).PCR products fractionated on agarose gels of UV-treated or *Xmn*I digested DNA (B).

Title: Changes in DNA damage, molecular integrity, and copy number for plastid DNA and mitochondrial DNA during maize development

Authors: Rachana A. Kumar, Delene J. Oldenburg and Arnold J. Bendich

Supplemental material 4

Statistical analysis

Maize seedlings were grown in three light conditions (dark, dark-to-light and light) and tissues representing four developmental stages (base of stalk; top of stalk; leaf1; and leaf2, see abbreviations in Table S2) were analyzed. Seedlings for five sets of independent biological replicates were grown and tissues harvested. A total of sixty ttDNA samples were produced (4 tissues x 3 growth conditions x 5 biological replicates). Statistical analyses were performed to compare the growth conditions (Table S3), the developmental stages (Table S4), growth conditions for each tissue (Table S5) and the tissues for each growth condition (Table S6). Statistical analyses were carried out in two steps using an R program. First, the ANOVA model was used to determine if there were any significant differences between growth conditions and among tissues. To satisfy the normality requirement for this model, the data were log-transformed prior to analysis. The second step was performed only if the first step showed significant differences. We used the Tukey multiple comparisons test (Tukey HSD) to evaluate those pairs of growth conditions or tissues or their combinations showing significant differences. For example, if ANOVA showed $p<0.05$ for growth conditions and tissues, then the Tukey HSD test was used to compare all the combinations for four tissues and three growth conditions. Abbreviations for tissues are given below. Color code: green for ptDNA; purple for mtDNA. ns: not significant. Significance codes: *** $p \leq 0.001$; ** $p \leq 0.01$; * $p \leq 0.05$.

Table S2 Abbreviations

Tissues	Abbreviation
Base of stalk	S1
Top of stalk	S2
Leaf2	L2
Leaf1	L1

Table S3 Tukey comparisons of growth conditions

Tissues compared	% unimpeded orgDNA	qPCR	miPCR	% unimpeded orgDNA	qPCR	miPCR
S1:S2	ns	***	**	ns	***	ns
S1:L2	*	***	ns	**	***	***
S1:L1	***	***	ns	***	***	***
S2:L2	*	***	ns	**	***	***
S2:L1	***	***	***	***	**	***
L2:L1	*	ns	**	ns	ns	ns

Table S4 Tukey comparisons of developmental stages

Growth conditions compared	% unimpeded orgDNA	qPCR	miPCR	% unimpeded orgDNA	qPCR	miPCR
Dark and Dark-to-light	ns	ns	*	ns	ns	ns
Dark and Light	**	ns	**	*	ns	*
Dark-to-light and Light	ns	ns	ns	ns	ns	ns

Table S5 Tukey multiple comparisons of growth conditions for each tissue

	% unimpeded orgDNA	qPCR	miPCR	% unimpeded orgDNA	qPCR	miPCR
**Dark and Dark-to-light**						
S1	**	ns	ns	*	ns	ns
S2	ns	ns	ns	ns	*	ns
L2	ns	ns	ns	ns	ns	ns
L1	ns	ns	ns	ns	*	ns
**Dark and Light**						
S1	***	ns	***	*	ns	**
S2	ns	ns	ns	ns	ns	ns
L2	*	ns	ns	ns	ns	ns
L1	ns	ns	ns	ns	***	ns
**Light and Dark-to-light**						
S1	ns	ns	ns	ns	ns	ns
S2	ns	ns	ns	ns	ns	ns
L2	ns	ns	ns	ns	ns	ns
L1	ns	ns	ns	ns	ns	ns

Table S6 Tukey multiple comparisons of tissues for each growth condition

	% unimpeded orgDNA	qPCR	miPCR	% unimpeded orgDNA	qPCR	miPCR
**Dark**						
S1:S2	*	***	ns	*	***	**
S1:L2	**	***	ns	**	***	*
S1:L1	***	***	ns	***	***	***
S2:L2	ns	ns	ns	ns	ns	ns
S2:L1	**	ns	*	*	ns	*
L2:L1	ns	ns	ns	*	ns	ns
**Dark-to-light**						
S1:S2	ns	***	ns	ns	***	ns
S1:L2	**	***	ns	*	***	*
S1:L1	**	***	ns	*	***	***
S2:L2	*	ns	ns	ns	ns	ns
S2:L1	**	ns	*	ns	ns	ns
L2:L1	ns	ns	ns	ns	ns	ns
**Light**						
S1:S2	ns	***	**	ns	**	ns
S1:L2	*	***	ns	ns	***	ns
S1:L1	ns	***	ns	ns	***	ns
S2:L2	**	*	ns	*	ns	*
S2:L1	*	ns	ns	*	*	*
L2:L1	ns	ns	ns	ns	ns	ns

Title: Changes in DNA damage, molecular integrity, and copy number for plastid DNA and mitochondrial DNA during maize development

Authors: Rachana A. Kumar, Delene J. Oldenburg and Arnold J. Bendich

Supplemental material 5

Regression analysis

Correlation between ptDNA and mtDNA

A linear (Fig. S11 to S16) or logarithmic (Fig. S17 and S18) regression model was employed to analyze the relationships between ptDNA and mtDNA for orgDNA impediments/10 kb and orgDNA copy number determined using qPCR, long PCR, and miPCR. For graphing, the averaged values across all the biological replicates for a given tissue were used. For example, five values from five biological replicates for base of the stalk grown in dark were averaged. A total of 12 values were obtained (4 tissues x 3 growth conditions) for ptDNA as well as for mtDNA. These values were used for regression analysis in Excel to give R^2 (coefficient of determination) and p value.

A strong correlation, with R^2 value close to 1 and $p<0.05$, indicate a tight relationship for a given parameter in both organelles. This correspondence may suggest a common DNA regulatory mechanism in both the organelles. For example, a strong correlation between ptDNA and mtDNA for orgDNA impediments may indicate that the DNA maintenance (damage and/or repair) pathways are operating in a similar way in both the organelles. Furthermore, a strong correlation may have a positive or a negative slope of line. A negative slope would indicate an inverse relationship, whereas a positive slope would be evidence of a direct relationship. For example, qPCR determined ptDNA and mtDNA copies show a strong correlation with a negative slope. This is because the ptDNA copies increase while mtDNA copies decrease during development. This result suggests a common mechanism for regulating orgDNA levels, but with a different outcome with respect to orgDNA retention.

Regression analysis may be used to determine an unknown value from a known value using a linear or logarithmic equation. For example, if there is a strong linear correlation with the equation $y=19x+1282$, there is a positive correlation between ptDNA/nucDNA (ordinate) and mtDNA/nucDNA (abscissa). If the mtDNA copies for a tissue are known (20, for example), then ptDNA copies can be calculated as 1662.

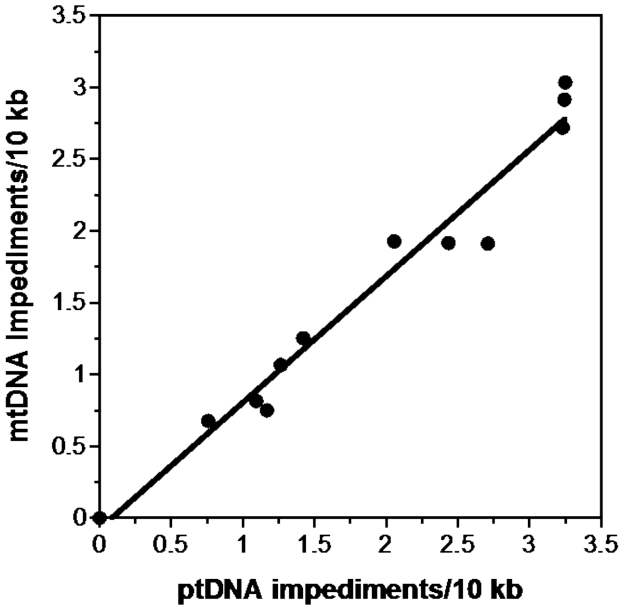

Fig. S11 Correlation between ptDNA and mtDNA for impediments/10 kb

The relationship between ptDNA and mtDNA was analyzed by the linear regression model. The average values for all the tissues and growth conditions were used to plot the graph. Analysis showed a strong correlation between ptDNA and mtDNA, with $R^2 = 0.97$ and $p = 1.3 \times 10^{-8}$.

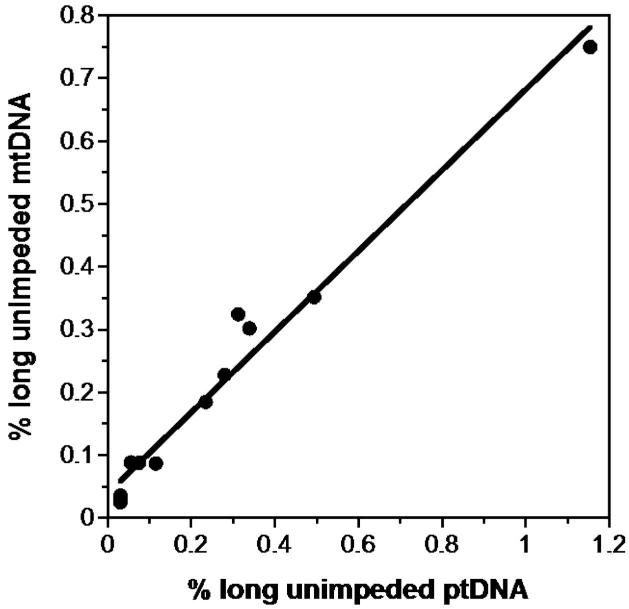

Fig. S12 Correlation between ptDNA and mtDNA for percentage of unimpeded long orgDNA copies

The relationship between ptDNA and mtDNA was analyzed by the linear regression model. The average values for all the tissues and growth conditions were used to plot the graph. Analysis showed a strong correlation between ptDNA and mtDNA, with $R^2 = 0.97$ and = 4.8×10^{-9}.

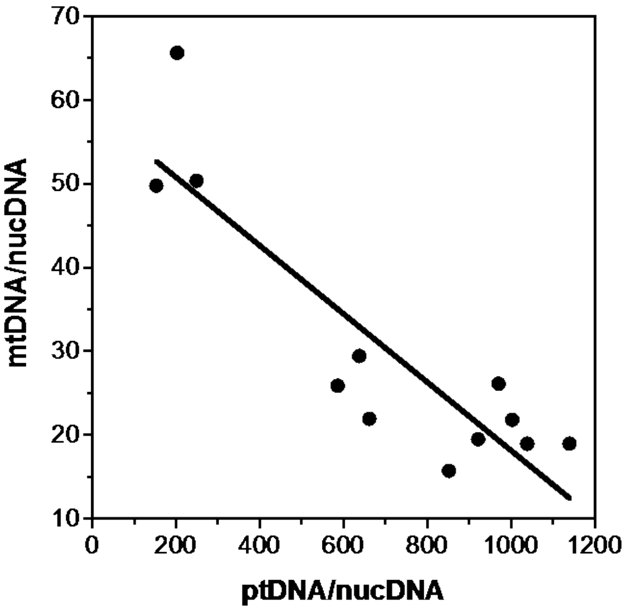

Fig. S13 Correlation between ptDNA/nucDNA and mtDNA/nucDNA determined by qPCR

The relationship between ptDNA and mtDNA was analyzed by the linear regression model. The average values for all the tissues and growth conditions were used. There is a strong correlation between ptDNA and mtDNA, with $R^2 = 0.8$ and $p = 1.4 \times 10^{-4}$.

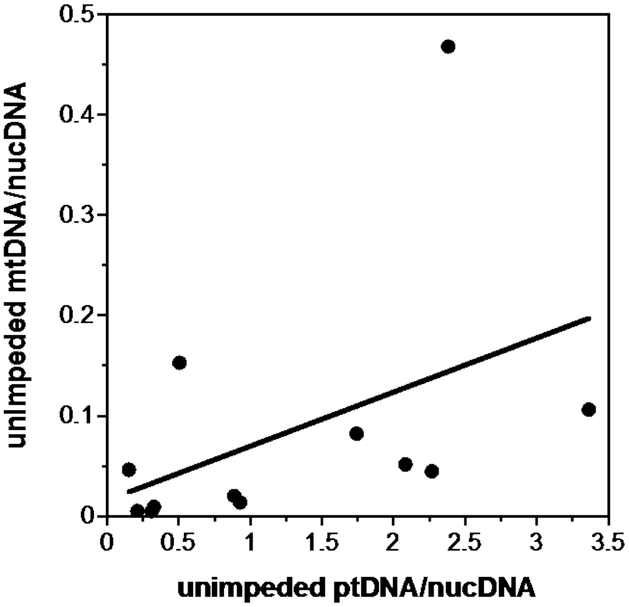

Fig. S14 Correlation between ptDNA and mtDNA for unimpeded orgDNA/nucDNA

The relationship between ptDNA and mtDNA was analyzed by the linear regression model. The average values for all the tissues and growth conditions were used to plot the graph. Analysis showed no correlation between ptDNA and mtDNA, with $R^2 = 0.2$ and $p = 0.14$.

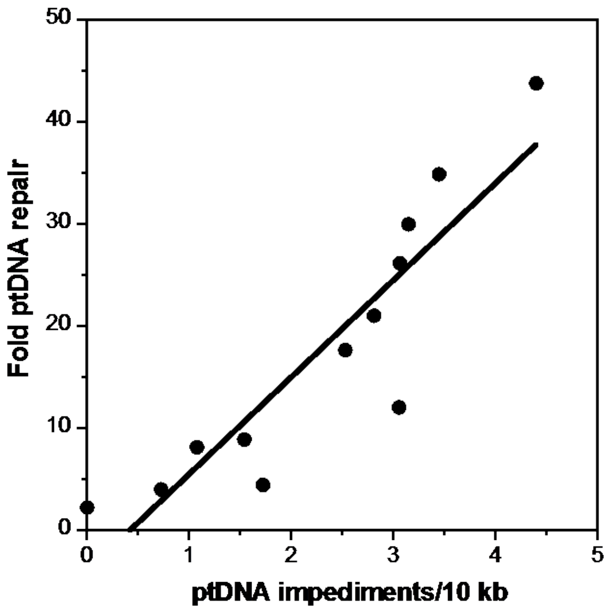

Fig. S15 Correlation between fold ptDNA repair and ptDNA impediments/10 kb

The relationship between amount of ptDNA damage and repair was analyzed by the linear regression model. The average values for all the tissues and growth conditions were used to plot the graph. Analysis showed a strong correlation between ptDNA damage and repair, with R^2 = 0.8 and p = 6.3 X 10^{-5}.

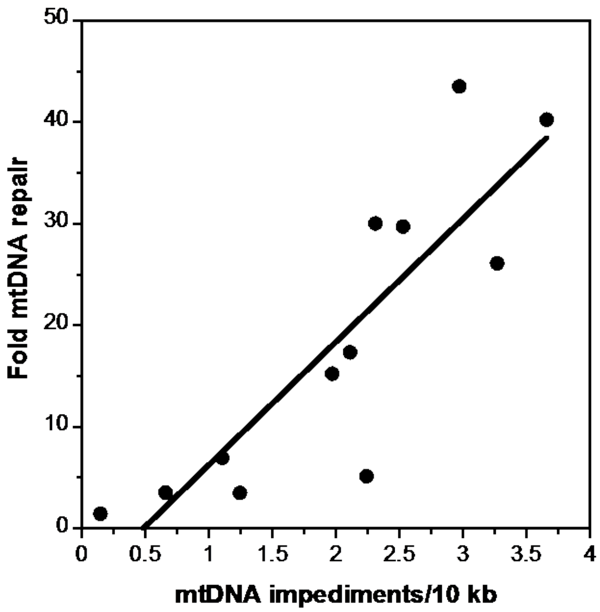

Fig. S16 Correlation between fold mtDNA repair and mtDNA impediments/10 kb

The relationship between amount of mtDNA damage and repair was analyzed by the linear regression model. The average values for all the tissues and growth conditions were used to plot the graph. Analysis showed a strong correlation between mtDNA damage and repair, with R^2 = 0.7 and p = 3.9,X,10^{-4}.

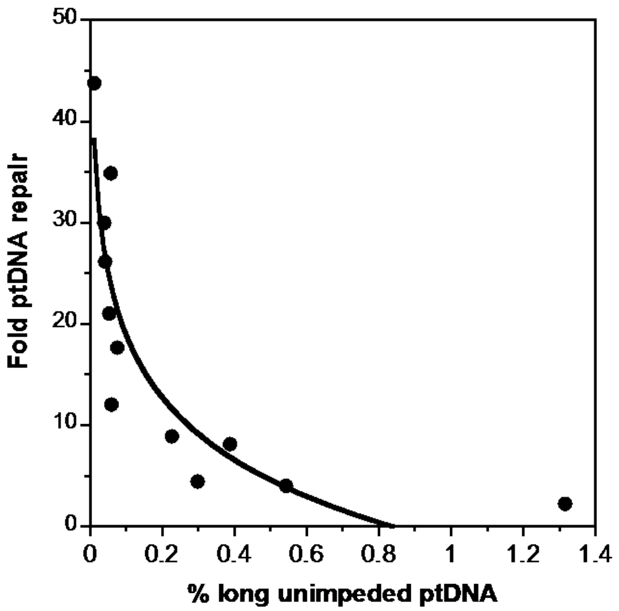

Fig. S17 Correlation between fold ptDNA repair and % unimpeded ptDNA

The relationship between amount of ptDNA repair and % unimpeded ptDNA was analyzed by the logarithmic regression model. The average values for all the tissues and growth conditions were used to plot the graph. Analysis showed an inverse correlation between ptDNA repair and % unimpeded ptDNA, with $R^2 = 0.81$ and $p = 0.02$.

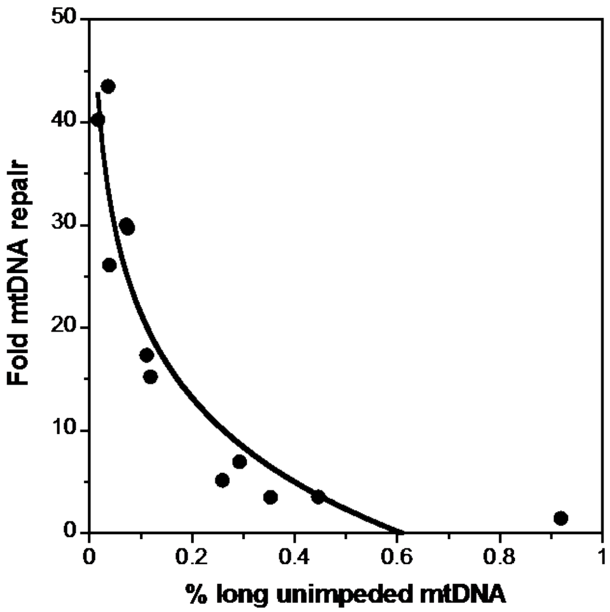

Fig. S18 Correlation between fold mtDNA repair and % unimpeded mtDNA

The relationship between amount of mtDNA repair and % unimpeded mtDNA was analyzed by the logarithmic regression model. The average values for all the tissues and growth conditions were used to plot the graph. Analysis showed an inverse correlation between mtDNA repair and % unimpeded mtDNA, with $R^2 = 0.88$ and $p = 5 \times 10^{-3}$.
